# High-resolution metagenomic reconstruction of the freshwater spring bloom

**DOI:** 10.1186/s40168-022-01451-4

**Published:** 2023-01-26

**Authors:** Vinicius S. Kavagutti, Paul-Adrian Bulzu, Cecilia M. Chiriac, Michaela M. Salcher, Indranil Mukherjee, Tanja Shabarova, Vesna Grujčić, Maliheh Mehrshad, Vojtěch Kasalický, Adrian-Stefan Andrei, Jitka Jezberová, Jaromir Seďa, Pavel Rychtecký, Petr Znachor, Karel Šimek, Rohit Ghai

**Affiliations:** 1 Institute of Hydrobiology, Biology Centre CAS, Na Sádkách 7, 370 05 České Budějovice, Czech Republic; 2 Faculty of Science, University of South Bohemia, Branišovská 31, 370 05 České Budějovice, Czech Republic; 3 Present address: Department of Ecology, Environment and Plant Sciences, Stockholm University, Stockholm, Sweden; 4 Present address: Department of Aquatic Sciences and Assessment, Swedish University of Agricultural Sciences, Uppsala, 750 07 Sweden; 5 Limnological Station, Microbial Evogenomics Lab (MiEL), University of Zurich, Kilchberg, Switzerland

## Abstract

**Background:**

The phytoplankton spring bloom in freshwater habitats is a complex, recurring, and dynamic ecological spectacle that unfolds at multiple biological scales. Although enormous taxonomic shifts in microbial assemblages during and after the bloom have been reported, genomic information on the microbial community of the spring bloom remains scarce.

**Results:**

We performed a high-resolution spatio-temporal sampling of the spring bloom in a freshwater reservoir and describe a multitude of previously unknown taxa using metagenome-assembled genomes of eukaryotes, prokaryotes, and viruses in combination with a broad array of methodologies. The recovered genomes reveal multiple distributional dynamics for several bacterial groups with progressively increasing stratification. Analyses of abundances of metagenome-assembled genomes in concert with CARD-FISH revealed remarkably similar in situ doubling time estimates for dominant genome-streamlined microbial lineages. Discordance between quantitations of cryptophytes arising from sequence data and microscopic identification suggested the presence of hidden, yet extremely abundant aplastidic cryptophytes that were confirmed by CARD-FISH analyses. Aplastidic cryptophytes are prevalent throughout the water column but have never been considered in prior models of plankton dynamics. We also recovered the first metagenomic-assembled genomes of freshwater protists (a diatom and a haptophyte) along with thousands of giant viral genomic contigs, some of which appeared similar to viruses infecting haptophytes but owing to lack of known representatives, most remained without any indication of their hosts. The contrasting distribution of giant viruses that are present in the entire water column to that of parasitic perkinsids residing largely in deeper waters allows us to propose giant viruses as the biological agents of top-down control and bloom collapse, likely in combination with bottom-up factors like a nutrient limitation.

**Conclusion:**

We reconstructed thousands of genomes of microbes and viruses from a freshwater spring bloom and show that such large-scale genome recovery allows tracking of planktonic succession in great detail. However, integration of metagenomic information with other methodologies (e.g., microscopy, CARD-FISH) remains critical to reveal diverse phenomena (e.g., distributional patterns, in situ doubling times) and novel participants (e.g., aplastidic cryptophytes) and to further refine existing ecological models (e.g., factors affecting bloom collapse). This work provides a genomic foundation for future approaches towards a fine-scale characterization of the organisms in relation to the rapidly changing environment during the course of the freshwater spring bloom.

Video Abstract

**Supplementary Information:**

The online version contains supplementary material available at 10.1186/s40168-022-01451-4.

## Introduction

The spring bloom in freshwaters is a fascinating and dynamic phenomenon that has captivated the attention of microbiologists for decades [1–3]. Typically, all water bodies in temperate regions experience low temperatures in winter and react to the onset of spring when a combined effect of multiple physicochemical parameters initiates a cascade of events leading to a drastic change in the resident microbial community [1, 2]. A classic spring bloom scenario unfolds by a mixing of the water column, after which increasing light, temperature, and nutrients allow for the development of a phytoplankton bloom (often dominated by cryptophytes and diatoms), characterized by a peak of chlorophyll-*a*. [1, 4–6]. Multiple factors like enhanced grazing by protists [7], mortality by viruses/parasites [8, 9], nutrient exhaustion [10], and increasing zooplankton predation [1] lead to the collapse of the bloom, giving way to the clear water phase when phytoplankton decline dramatically. These changes in plankton assemblages have been encapsulated into generalized models that incorporate multiple factors affecting the initiation, expansion, and collapse of the spring bloom and the development of the clear water phase, e.g., the Plankton Ecology Group (PEG) model [1, 2].

It has been argued that capturing such events with a fast turnover of the community in a matter of a few weeks necessitates a high-temporal sampling approach, i.e., sampling every 2/3 days to identify short-lived peaks of rapidly growing microbes (generation times in hours to days) as seasonal (e.g., spring, summer, winter) or monthly samplings are insufficient [6, 11]. While long-term time series exploration of freshwater habitats is becoming increasingly common, and approaches ranging from amplicon analyses [12–14], fluorescence in situ hybridization and catalyzed reporter deposition (CARD-FISH) [15–17], and cultivation [18] to metagenomic sequencing [19, 20] have been applied, studies with high-temporal sampling, particularly in freshwaters are limited [4–6, 21, 22]. Even so, many approaches have been applied to disentangle this complex phenomenon. Spring blooms in freshwaters have been investigated by using CARD-FISH probes to detect short-lived peaks in multiple bacterial groups to couple their abundances to dynamics of phytoplankton and zooplankton [6], to follow specific bacterial groups (e.g., Flavobacteria) [4, 5, 18], cryptophytes [23], or ciliates [24, 25]. Other studies have addressed the uptake of specific metabolites (Chitin, NAG) [5, 26]. Similar algal-microbial blooms were also investigated in marine habitats, which has brought new important insights into factors affecting succession, e.g., availability of diverse carbohydrates or phosphorus [27, 28] and promoted cultivation efforts targeting fast-responding heterotrophs like Bacteroidota, Alphaproteobacteria, Gammaproteobacteria, and Verrucomicrobiota [29, 30].

However, there are methodological limitations of many approaches that have been previously applied that preclude the discovery of novel participants of the spring bloom across the entire community. For instance, CARD-FISH analyses require the design of probes that target specific lineages, which may not be possible for many taxonomic groups of protists [31]. Similarly, amplicon-based studies provide no information on viruses, and culture-based approaches are biased towards relatively easily cultivable copiotrophs (e.g., Flavobacteria), overlooking many dominant, genome-streamlined oligotrophic microbes that remain hard to culture [32, 33]. Finally, microscopic analyses alone are insufficient to reliably distinguish many heterotrophic nanoflagellates [34]. In this regard, a metagenomic approach towards the de novo recovery of genomes of novel or understudied groups has been singularly lacking. Such an approach can potentially reveal genomes of prokaryotes, eukaryotes, and viruses, providing a much higher resolution of the entire community. This work presents a high-resolution temporal dissection of the annual spring bloom in a freshwater reservoir over 37 days of intensive sampling (every 2–3 days, a total of 57 samples), combining classical microbiological methods, CARD-FISH analyses, and metagenomic sequencing of three different size fractions. This allowed the recovery of metagenome-assembled genomes of prokaryotes, eukaryotes, and their viruses that constitute the overall microbial community. Moreover, sampling the hypolimnion that is typically ignored provides a more complete perspective of the entire water column. The application of a high-frequency sampling coupled with a metagenomic approach allowed us to add significant detail to the overall tapestry of planktonic succession in this remarkable event.

## Results and discussion

### Initial characterization of the spring bloom

**Fig. 1:**
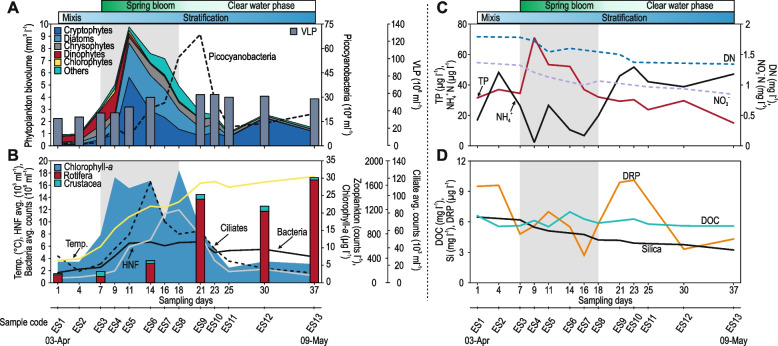
Time course of different features of the spring bloom in the epilimnion. **A** Phytoplankton biovolume, picocyanobacterial abundances, and viral-like particle counts (VLP). **B** The concentration of chlorophyll-*a*, temperature, counts of total heterotrophic bacteria, heterotrophic nanoflagellates (HNF), ciliates, rotifers, and crustaceans. **C** Total phosphorus (TP), dissolved nitrogen (DN), nitrate and ammonium concentrations, and **D** dissolved reactive phosphorus (DRP), dissolved organic carbon (DOC), and silica concentrations. The gray rectangle in the background shows the duration of the spring bloom

To capture the dynamic events of the spring bloom, a high-frequency sampling approach was implemented, sampling the epilimnion every 2–3 days (from Apr 03 to May 09, 2018) and the hypolimnion every week (see the “Methods” section for details). The chief features are summarized in Fig. 1 (epilimnion) and Additional file 1: Figure S1 (hypolimnion). The present study time frame can be divided into two distinct phases based on the water mixing patterns, chlorophyll-*a* dynamics, and the composition of the planktonic community. The first stage is the mixis (days 1–4), when the entire water column is vertically isothermal (4 °C), with low chlorophyll-*a* (5 μg l^−1^), and high nutrient (dissolved inorganic nitrogen and phosphorus) concentrations. The second is characterized by the progressive stratification induced by increased temperature (days 7–37). Two major events mark the second stage. The spring bloom becomes evident with high measurements of chlorophyll-*a* (days 7–18). Phytoplankton started to increase between days 4 and 7, peaking at day 18 (chlorophyll-*a* concentration of 30 μg l^−1^). Finally, the clear water phase (days 30–37) becomes distinguishable by a well-established water density gradient, low chlorophyll values, and nutrient depletion, i.e., phosphorus. According to microscopic observations, diatoms and photosynthetic cryptophytes accounted for most phytoplankton biomass during the bloom period (days 7–18). A number of diverse diatom genera, e.g., *Asterionella*, *Fragilaria*, *Nitzschia*, *Synedra*, and centric diatoms (potentially *Cyclotella*) prevailed, in addition to the mixotrophic haptophyte *Chrysochromulina* (Additional file 2: Table S1). A small but discernible maximum in cryptophytes was also observed around day 30 (Fig. 1).

The highest concentration of total phosphorus (TP) was paralleled with the lowest availability of DRP (dissolved reactive phosphorus) during the bloom, reflecting a fast turnover of dissolved inorganic phosphorus and its accumulation by phytoplankton. Ammonium was rapidly depleted owing to uptake by phytoplankton as reduced nitrogen forms are preferentially taken up, and if not available, phytoplankton utilizes nitrate as an alternative nitrogen source [35]. Nitrate also decreased gradually during the study period. Upon bloom-collapse, ammonium and DRP were released from phytoplankton by excretion and sloppy feeding of zooplankton [36–38]. Total heterotrophic prokaryotic numbers rapidly increased from 2 to 6 × 10^6^ cells ml^−1^ during the phytoplankton bloom and remained relatively stable thereafter. Picocyanobacteria reached their highest levels after the first bloom event (day 21). However, they accounted for only a negligible portion of the total phytoplankton biomass (maximum 7 × 10^4^ cells ml ^−1^). Ciliates reached their maximum on day 14, preceding a peak of HNF (heterotrophic nanoflagellates) by 4 days. The abundance of zooplankton (rotifers and crustaceans) continuously increased and reached the highest levels in the clear water phase (Fig. 1, Additional file 1: Figure S1).

Despite ciliates and HNF being important consumers of prokaryotes in aquatic habitats, their temporal dynamics are hard to capture since they often form short-lived peaks, necessitating a high-frequency sampling approach [4–6]. In this study, we detected peaks for both groups during the spring bloom (Fig. 1, Additional file 1: Figure S2). Estimates of total grazing rates suggest that HNF are more important bacterivores than ciliates during this period (Additional file 1: Figure S2), which has been observed before [6]. There are also clear shifts in the ciliate populations, from those that feed largely on algae, HNF, and cryptophytes (e.g., *Balanion*, *Rimostrombidium*, *Urotricha*) [24, 39], towards filter-feeding omnivores (e.g., *Halteria*) capable of feeding on multiple food sources in a size range from small bacteria to small algae and HNF [24, 40].

### A metagenomic perspective of the spring bloom

**Fig. 2:**
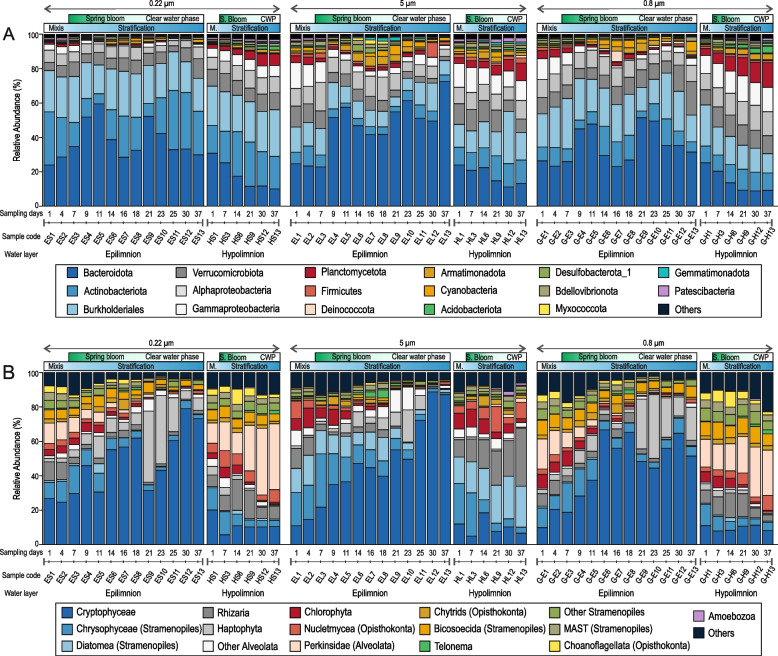
Relative abundances of different taxonomic groups (expressed as a percentage) in the spring bloom as assessed by 16S rRNA (top, prokaryotes) and 18S rRNA (below, eukaryotes) gene read sequences. The results from different filters are shown (left, 0.22 μm; middle, 5 μm and right, 0.8 μm). Epilimnion and hypolimnion samples are also indicated

We analyzed 57 metagenomic samples from two different depths over 37 days (a total of 1.96 billion reads, ca. 830 Gb), using multiple filter sizes (5 μm, 0.8 μm, and 0.22 μm) and different filtration methods (see Methods) providing an unprecedented view of the changes in the microbial community across time (Fig. 2). 16S rRNA screening of the 0.22-μm filters indicated that Actinobacteriota, Bacteroidota, and Burkholderiales (formerly Betaproteobacteria) are the most dominant in the epilimnion during the entire period, followed by Alphaproteobacteria and Verrucomicrobiota (Fig. 2). However, even at this broad taxonomic level, distinct responses of these taxa are clearly discernible. Bacteroidota rapidly increased to > 50% of 16S rRNA reads at the onset of the phytoplankton bloom (days 9–11), slightly decreased thereafter, and showed a second maximum at day 21 when the bloom started to decay. These bacteria are likely relatively large or associated with protists or aggregates [41], as is evident from their higher recovery from the 5-μm filter. The maximum abundance of Bacteroidota happened in parallel with two sharp decreases in the relative abundance of Actinobacteriota (belonging mostly to the order ‘*Ca.* Nanopelagicales’) detected in the epilimnion on days 11 and 21, followed by their recovery to previous levels (Fig. 1). Freshwater Actinobacteriota are repeatedly reported to be among the most dominant microbial groups across various freshwater habitats, including this particular site [20, 32, 42, 43]. Opposed to Bacteroidota, they were much less recovered from 5-μm and 0.8-μm filters because of their small size. On the other hand, sequences affiliated with Burkholderiales appeared to be relatively stable throughout the sampling period. A continuous increase and a preference for the hypolimnion in certain taxa is visible for Gammaproteobacteria and Planctomycetota (especially on the 0.8-μm filter). Planctomycetota have been previously shown to prefer hypolimnetic waters and appear in the upper layers mainly when the stratification is eroded [44]. A similar trend was observed here, with their numbers continuously decreasing in the epilimnion with rising temperature and gradually increasing in the hypolimnion. Surprisingly, we also observed an increase in Armatimonadota in the hypolimnion, a group scarcely reported from freshwaters. All other microbial groups remain at lower levels except for small increases in Deinococcota captured on the 0.8-μm gravity filters in the epilimnion on day 16.

Similar to the trends observed for (plastidic) cryptophytes in microscopy (Fig. 1), cryptophyte rRNA gene sequences represent the major part of all eukaryotic sequences recovered from the epilimnion (Fig. 2). At the same time, their abundances are considerably lower in the hypolimnion. Both cryptophyte peaks that were observed microscopically (Fig. 1) were also detected in the rRNA gene abundance analysis (Fig. 2). Across all filter sizes, and in both epilimnion and hypolimnion, Chrysophyceae and Chlorophyta sequences appeared to decline continuously, and Rhizaria (SAR group) sequences were more abundant in the hypolimnion.

Short-lived peaks in other groups were also observed. For example, two peaks of haptophytes were visible in the epilimnion on days 11 and 21–25 (Fig. 2). These blooms were mostly composed of the mixotroph *Chrysochromulina* (Prymnesiophyceae), as we also recovered 19 metagenomic bins that could be ascribed to *Chrysochromulina* (see below). *Chrysochromulina* was also observed in the samples accounting for 1–15% of the total phytoplankton biovolume in the later stages of the bloom. Another consistent feature of the bloom was the appearance of diatoms (confirmed by microscopy) in the first phase of the bloom (Fig. 1, Additional file 2: Table S1). The rRNA sequences of diatoms could be mostly ascribed to *Thalassiosira* and corroborated by the recovery of multiple partial genomes of *Thalassiosira* from the metagenomic data (see below). Other photosynthetic protists, e.g., Chlorophyta, continuously decrease in abundance as the bloom progresses (Fig. 2).

Most studies on the freshwater spring bloom have focussed primarily on the epilimnion [4–6, 26] because photosynthetic phytoplankton has been considered the major players. The changes in the microbial community in deeper water layers have remained relatively unknown. Remarkably, the abundance of sequences ascribed to Perkinsozoa (a poorly understood group of parasitic protozoa) increased in the hypolimnion and decreased in the epilimnion. Conceivably, a low-temperature preference for Perkinsozoa might exist, although their extremely high relative abundances (ca. 40%) in the hypolimnion (Fig. 2) suggest there may be other unknown driving factors as well. It remains unclear if these Perkinsozoa are parasitic or free-living. Parasitic Perkinsozoa are better known for marine habitats and infect mollusks [45], dinoflagellates [46], chlorophytes [47], and even fish [48]. They have also been reported from surface freshwaters [15, 49, 50] and deeper water strata [51, 52]. Some perkinsids have even been described to infect cryptophytes (e.g., the perkinsid *Cryptophagus* infecting the cryptophyte *Chilomonas*) [53]. Given their broad host range in the freshwaters and the eukaryotic community’s complexity, their preferred freshwater hosts remain obscure. In case they infect cryptophytes, which represent a prominent segment of the spring phytoplankton bloom, the abundant host population would also provide a niche for the rapid proliferation of the parasites. However, as perkinsids are far more abundant in the hypolimnion than in the epilimnion, it is unlikely that they affect bloom collapse significantly in the epilimnion where the bloom takes place. Moreover, the viral collapse of marine algal blooms is well-known [54]. In support of this hypothesis, the metagenomic data suggest a greater abundance of perkinsids in the hypolimnion but not in the epilimnion. On the other hand, eukaryotic viruses are prevalent throughout the water column and likely contribute more to the bloom collapse in the epilimnion in concert with other bottom-up controls e.g., nutrient limitations (see also below).

### Recovery of bacterial and phage genomes

A total of 2214 bacterial metagenome-assembled genomes (MAGs) (≥ 40% completeness and ≤ 5% contamination) were reconstructed from 57 metagenomic datasets generated from three different filter-pore sizes (0.22 μm, 0.8 μm, 5 μm) and two different depths. See Additional file 3: Table S2 and Additional file 1: Figure S3 for more details. The number recovered from each filter varies greatly between fractions, 1288 from 0.22 μm, 808 from 0.8 μm, and only 112 from the 5 μm filter, i.e., increasing filter-pore size reduces prokaryotic genome recovery dramatically as they likely capture more eukaryotic genomic material. The most recovered bacterial MAGs were from the phyla Bacteroidota (*n* = 690), Proteobacteriota (*n* = 677: 493 Gammaproteobacteria and 184 Alphaproteobacteria), Actinobacteriota (*n* = 453), Verrucomicrobiota (*n* = 196), and Planctomycetota (*n* = 92) (See also Additional file 3: Table S2). De-replication of these MAGs resulted in 855 genomes representing prokaryotic bacterial diversity along the spring bloom (Additional file 3: Table S2). We did not recover any archaeal MAGs from these datasets.

It is worth noting that 615 MAGs encode putative rhodopsins distributed across 326 clusters of very diverse phyla, i.e., Actinobacteriota, Bacteroidota, Chloroflexota, Proteobacteria, and Verrucomicrobiota. Most encode type-1 rhodopsin (*n* = 508, 223 clusters) and a smaller number of heliorhodopsin (*n* = 214, 103 clusters). Moreover, 203 of those MAGs encode both types of rhodopsin (48 clusters). In sum, nearly 38% of the microbial community (326 out of 855 dereplicated MAGs) responds to light in a rhodopsin-dependent fashion (Additional file 4: Table S3). Almost all these type-1 rhodopsins across different phyla display the DTE motif in transmembrane helix 3, which suggests all these are proteorhodopsins [55], enabling outward proton translocation to generate the proton-motive force to produce ATP. Only a few exceptions to this were found, with less than ten sequences with DTG and ATI motifs that also indicate outward H^+^ pumping activity. Moreover, all detected rhodopsins were found to be green-absorbing rhodopsins (leucine as residue 93, bacteriorhodopsin numbering) [56], except for two sequences originating from freshwater ‘*Ca.* Nanopelagicales’ MAGs appeared to be blue-light absorbing (glutamine as residue 93). It has been suggested before that as blue light penetrates deeper into the water column, organisms encoding blue-light-absorbing rhodopsins might have an advantage in deeper waters [57]. These two MAGs belong to two clades that are basal to *‘Ca*. Nanopelagicus’ and *‘Ca*. Planktophila’ and are found at low abundances at the mixis phase and later transition to deeper waters where the possession of blue-light absorbing rhodopsin may provide a selective advantage. Other MAGs related to these possess green-light-absorbing rhodopsins.

While viruses are recognized as important participants in the spring bloom, no freshwater viruses have been isolated, nor have any viral genomes recovered from this specific period. We recovered 679 complete phage genomes (see the “Methods” section) from the three different filter types that were dereplicated to 175 complete phage genomes. Previously, we have published a large dataset of complete freshwater phage genomes [20], and 1398 genomes originated from the same site (Římov phages). De-replication of all phage genomes from the same site in these two datasets together revealed that of the 679 phage genomes recovered in this study, 129 had already been seen before and may be considered ‘persistent’ dwellers in the environment for at least 2 years. The remaining 462 (dereplicated 162) are novel phage genomes identified in this work. We identified 35 genomes (out of 679) that were predicted to infect freshwater Actinobacteriota. Actinophages can be recognized because they encode an actinobacteriota-specific transcription factor, *whiB *[58]. Only three of these 35 phages were described before [20]. We also recovered six complete phage genomes (30–31 Kb, three dereplicated clusters) that are predicted to infect freshwater cyanobacteria. All of these encodes a cyanobacterial-specific RNA polymerase sigma factor (TIGR02997). This gene is largely specific to cyanobacteria (99% of all hits in GTDB are cyanobacterial) [59], strongly suggesting these are freshwater cyanophages. It has been shown before that similar sigma factors carried by cyanophages strongly repress cyanobacterial growth [60], and given that no photosystem genes were found in these phage genomes, this gene is likely important for the phage infection cycle. In further support of these being cyanophages, the metagenomic abundances of two of these predicted representative cyanophage genomes (HRS-EL5-C113 and HRSG-E8-C80, Additional file 1: Figure S4) also appeared to match the peak of picocyanobacteria by microscopy (time points ES9 and ES10, Fig. 1). Most freshwater cyanophage genomes from cultured isolates do not encode photosystem genes. Of the 18 currently available genomes, only two encode photosystems (both infecting *Synechococcus*), indicating that capturing photosystems for the phage infection cycle is not very common for freshwater cyanophages and other strategies may be more prevalent (Additional file 5: Table S4). Another seven phage genomes were predicted to infect Burkholderiales genomes using matches from tRNA genes (see methods) (Additional file 5: Table S4).

### Dynamics of selected, abundant prokaryotic groups

**Fig. 3:**
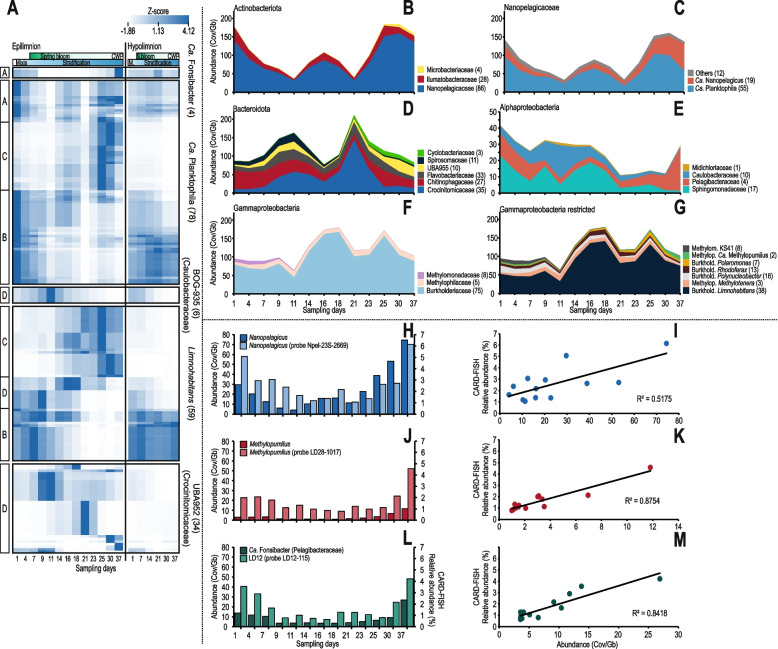
**A** Relative abundance of selected dereplicated MAGs during the spring bloom in the small filter (0.22 µm). Each row was normalized by *Z* score and clustered by average linkage (with Spearman’s rank correlation method). Different letters represent different patterns of abundance common within groups. The numbers of genomes used for each group are mentioned in parentheses. For more details about other groups, check Additional file 1: Figure S4, S5, and S6. **B**–**G** Abundances of selected microbial genomes/groups during the spring bloom: The *X*-axes show the progression of the bloom (in days), and *Y*-axes show the abundance of the MAGs. All *Y*-axes range from 0 to 200 except for Alphaproteobacteria (0–50). The numbers of dereplicated genomes used for each group are given in parentheses. Methylomonadaceae, Methylophilaceae, and Burkholderiaceae are abbreviated as ‘Methylom.’, ‘Methylop.’, and ‘Burkhold.’ respectively, in the figure. **H**–**M** Metagenomic abundance vs CARD-FISH relative abundance. **H**, **J**, and **I** Coverage per Gb on the left *Y*-axis and CARD-FISH relative abundance % on right *Y*-axis. **I**, **K**, and **M** Correlation between metagenomic abundance vs CARD-FISH relative abundance

Above all, the spring bloom is a transitory period for microbial populations that are directly affected by the increase in light availability and temperature and the onset of stratification coupled with rapid changes in the availability of nutrients [2]. This results in distinct microbial communities at both ends of the spectrum. The availability of MAGs from the entire spring period allows us to distinguish distinct modes of succession in the spring bloom at a genome level (Fig. 3 and Additional file 1: Figure S5 ~ S7). Temperature dependence is visible for several bacterial populations and phages (Fig. 3, Additional file 1: Figure S4, S8 ~ S12). At least four broad categories of populations are detectable during the spring period, (A) temperature generalists that are abundant both at the beginning and the end of the study period, perhaps with some fluctuations during the bloom, likely well adapted to both cold and warm temperatures (e.g., ‘*Ca.* Fonsibacter’, some ‘*Ca.* Planktophila’); (B) populations preferring colder temperatures that are primarily abundant in the epilimnion in the beginning but transition to the hypolimnion as surface temperature rises (some ‘*Ca.* Planktophila’ and *Limnohabitans*); (C) populations preferring warmer temperature, gradually reaching maxima in the clear water phase and not abundant in the hypolimnion (some ‘*Ca.* Planktophila’ and *Limnohabitans*); and finally (D) populations that also prefer warmer temperatures, but show sporadic, short-lived peaks in the epilimnion and similarly to (C) are not abundant in the hypolimnion (BOG935, UBA952, and some *Limnohabitans*). Populations D and A can broadly be categorized as bloom specialists and bloom avoiders, respectively.

It is also apparent that phylogenetically closely related genomes may have different ecological behavior, suggesting that minor genomic alterations may have significant ecological consequences (Additional file 1: Figure S5 ~ S7). The genome-streamlined Actinobacteriota represented by ‘*Ca.* Planktophila’ (‘*Ca.* Nanopelagicales’) appear to consist of very diverse populations displaying different dynamics during the spring period. Moreover, the populations in the epilimnion at the beginning of the spring bloom are quite different from those at the end, and groups that show similar abundance profiles are not necessarily phylogenetically related (Fig. 3, Additional file 1: Figure S6). Remarkably similar population replacement patterns in the epilimnion and transitions to the hypolimnion can also be seen for the faster-growing copiotroph *Limnohabitans* (Additional file 1: Figure S7). A high (micro-)diversity with different habitat preferences and organic matter utilization has been proposed for both taxa [32, 61, 62]. The opposite holds true for genome-streamlined Alphaproteobacteria represented by ‘*Ca.* Fonsibacter’ (four de-replicated genomes), where the abundances of genomes across the timeline are very similar with only minor differences. Their abundance declines with increasing chlorophyll and total phosphorus and remains low until the same populations recover during the clear water phase. The same clades also appear in the hypolimnion, and a clear-cut temperature dependence is not discernable. This suggests an intriguing reason for the success of ‘*Ca.* Fonsibacter’ in freshwaters in a low-chlorophyll regime: there is low overall genomic diversity (Additional file 1: Figure S4), but this low diversity is well adapted to variations in temperature [14, 63, 64]. This contrasts with most ‘*Ca.* Planktophila,’ where genomic diversity is high (Additional file 1: Figure S5), and separate clades usually have different temperature preferences. At the same time, only a few populations are equally abundant at the beginning and the end of the spring period.

Fine-scale dynamics of different taxa have been examined during the spring bloom using CARD-FISH, e.g., ‘*Ca.* Nanopelagicales’ (formerly acI Actinobacteria), *Limnohabitans*, and some lineages of Bacteroidota [4–6], but never coupled with a metagenomic assessment (i.e., MAGs) what allows a much finer distinction even between closely related organisms than is possible by CARD-FISH alone. For Actinobacteriota, as has been stated before, most MAGs were affiliated to ‘*Ca.* Nanopelagicales’ and, to a lesser extent Illumatobacteraceae (formerly acIV Actinobacteria) and Microbacteriaceae (Fig. 3). In the epilimnion, there is a pronounced minimum in the abundance of actinobacterial MAGs on day 11, a recovery until day 16, and another decline on day 21, followed by a recovery until day 25. The population decreases in ‘*Ca.* Nanopelagicales’ can be more specifically attributed to ‘*Ca.* Plankophila’, while the related genus, ‘*Ca.* Nanopelagicus’, which is generally more stable throughout, starts increasing in the clear water phase (days 30–37). A similar trend with sharp declines and rapid growth thereafter is also visible for Burkholderiaceae (mainly *Limnohabitans*, day 11) and Sphingomonadaceae (days 7 and 11, Fig. 3). However, it is unclear if these population collapses were caused by phage infection, grazing by protists, or competitive disadvantages to fast-growing copiotrophs. At the same time, other groups dramatically increase in numbers, e.g., Caulobacteraceae (at day 11), Crocinibacteraceae (days 11 and 23), and to a lesser extent, Flavobacteriaceae (day 9). Caulobacteraceae (genus BOG935), Crocinibacteraceae (genus UBA952), and Flavobacteriaceae appear to be bloom specialists, dominating only for a short period during favorable conditions. Similar typical algal bloom specialists have been observed before (e.g., *Fluviicola*, *Flavobacterium*) [4]; however, BOG935 and UBA952 represent new additions to this category of fast-growing copiotrophs, but no cultured representatives are as yet available. Also apparent is the relatively fast growth of *Limnohabitans* after a minimum at day 11, after which their abundance appears to stabilize. Other characteristic patterns are also visible, e.g., ‘*Ca.* Fonsibacter’ (shown as Pelagibacteriaceae in Fig. 3) has a pronounced minimum during the bloom; it is well known that these oligotrophs start growing in the clear water phase and reach their annual maximum in summer-autumn [16].

These relatively fast changes in multiple populations prompted us to examine the possibility of estimating in-situ growth rates for at least some taxa using metagenomic abundance data, especially for genome-streamlined microbes (‘*Ca.* Nanopelagicus’, *‘Ca.* Fonsibacter’, and ‘*Ca.* Methylopumilus’). While representatives of these taxa are available in culture [17, 65, 66], their doubling times in the natural environment remain unknown, except for a doubling time estimate made collectively for *‘Ca* Nanopelagicales’ (formerly acI Actinobacteria) [32], ca. 66 h based on CARD-FISH abundances [6]. Moreover, while some of these microbes have been obtained in culture, doubling time estimates from cultures might not be similar to those in real environmental conditions [67] where there is significant competition for resources, mortality by grazers (e.g., heterotrophic nanoflagellates) and viral infections. We also performed CARD-FISH on these selected genome-streamlined microbes throughout the spring bloom, using general probes for ‘*Ca.* Nanopelagicales’ and Methylophilaceae, and specific probes for the genera ‘*Ca.* Nanopelagicus’, ‘*Ca.* Fonsibacter’, and ‘*Ca.* Methylopumilus’ (See Additional file 6: Table S5). A high concordance was observed between the relative abundances in metagenomes, and CARD-FISH for all three genome-streamlined taxa examined (Fig. 3). Because mortality rate due to protists and viral infections is hard to predict without predation experiments, we used a value of 55% mortality rate for all bacterial groups (on average, 24% from grazing and 31% from viral infections) obtained from 19 temperate lakes [68]. Lastly, we also applied a codon-bias-based doubling time estimation from genomes using gRodon [69].

In the case of ‘*Ca.* Nanopelagicales’, Methylophilaceae, and ‘*Ca.* Fonsibacter’, there is broad agreement between doubling time estimates from recruitment and CARD-FISH (Table 1, Fig. 3), while results for ‘*Ca.* Nanopelagicus’ and ‘*Ca.* Methylopumilus’ differed by about two-fold. Generally, there appears more concordance at the higher taxonomic level than at the genus level. For all microbes, gRodon predicted a doubling time of > 5 h, which only suggests that the microbe is slow-growing, and the results are considered to be an underestimation. The observed doubling times for ‘*Ca.* Nanopelagicales’ (72 h) are remarkably similar to those previously reported (66 h) [6]. Upon correction, for mortality, this value was reduced to 37 h. Estimated doubling times for ‘*Ca.* Nanopelagicales’ and Methylophilaceae (using CARD-FISH or metagenomic abundance) fall in a similar range of 28–44 h. Surprisingly, similar estimates have been obtained for cultures of ‘*Ca.* Methylopumilus’ (0.4 divisions per day) [17] and ‘*Ca.* Fonsibacter’ (0.52 divisions per day) [65], and even for marine *Pelagibacter ubique* (0.4–0.58 divisions per day) [70], which translates to a doubling time of roughly 2 days, not very different from the values obtained here in the presence of mortality. This suggests that existing culture media for genome-streamlined microbes are already well-optimized. As is evident from the variations in doubling times computed at different sampling times for all microbial groups, the accuracy of such estimates may be influenced by environmental conditions and even the mode of observation. Still, they do provide a more realistic appreciation of the growth of highly abundant and cosmopolitan microbes in the natural environment.

**Table 1:** Observed and estimated doubling times in hours (h) (± SD) for genome-streamlined, oligotrophic taxa obtained using different approaches. The number of MAGs used for each group is indicated inside the brackets

	*Doubling times*				
	CARD-FISH		Metagenomic abundance		gRodon
Group	Observed, in situ^a^ (h)	Estimated^b^ (h)	Observed, in situ^a^ (h)	Estimated^b^ (h)	Inferred from genome (h)
*Ca.* Nanopelagicales (41)	72.12 ± 16.68	36.67 ± 4.15	44.44 ± 7.69	27.86 ± 3.18	10.67 ± 7.15
*Ca.* Nanopelagicus (17)	106.72 ± 72.72	56.06 ± 40.81	46.85 ± 3.16	28.94 ± 1.31	9.21 ± 2.43
Methylophilaceae (5)	87.85 ± 35.01	43.43 ± 14.03	80.1 ± 17.17	44.2 ± 9.34	11.2 ± 11.21
*Ca.* Methylopumilus (2)	134.74 ± 63.17	70.39 ± 33.3	61.66 ± 1.67	34 ± 0.5	22.98 ± 4.26
*Ca.* Fonsibacter (3)	114.66 ± 113.2	62.99 ± 51.01	106.78 ± 10.58	76.07 ± 5.47	31.12 ± 15.61

^a^Observed, in situ: average of in situ doubling times (in hours) obtained using observed abundances (assuming mortality)

^b^Estimated: average of estimated doubling times (in hours) obtained after correcting for 55% mortality

### Recovery of eukaryotic and giant viral genomes

Owing to the genomic complexity and large genome sizes, eukaryotic genomes have rarely been recovered from metagenomes, and the only recent report is from the marine habitat [71]. Upon examining the recovered MAGs after binning, we noticed that 25 appeared to originate from eukaryotes. Of these, 19 were highly related to *Chrysochromulina* (a Haptophyta) and four to *Thalassiosira* (a centric diatom). Genome completeness estimates using eukaryotic or lineage-specific markers (wherever identifiable) show completeness in the range of 6–37% (See Additional file 7: Table S6). Apart from *Chrysochromulina* and *Thalassiosira*, two other bins appeared to be affiliated to stramenopiles and cryptophyta, but as their genome completeness was only ca. 10%, they were not examined further. The recovered MAGs of *Chrysochromulina* and *Thalassiosira* represent the first metagenome-assembled genomes from freshwater eukaryotes.

**Fig. 4:**
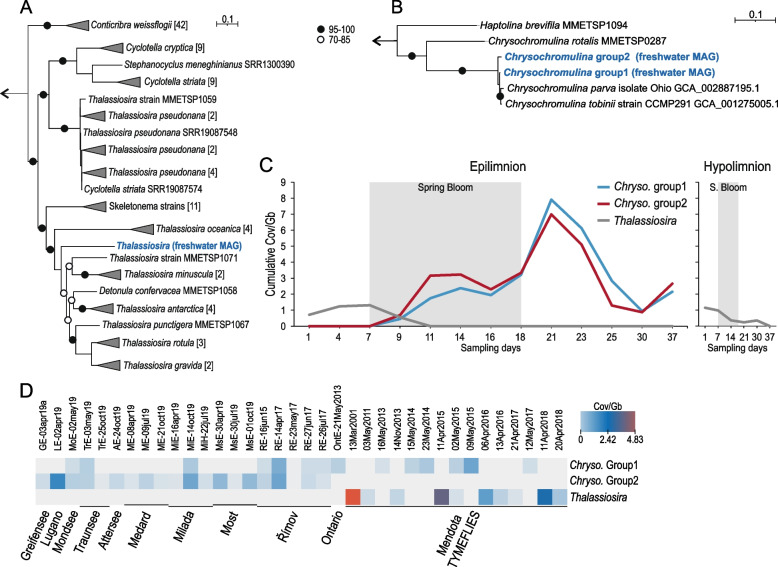
**A** Phylogenomic trees of *Thalassiosira.***B***Chrysochromulina* metagenome-assembled genomes. Ultrafast bootstrap values (UFB) are defined by full (95–100) and empty (70–85) circles. Collapsed clades (triangles) followed by square brackets containing the total number of members within the clade. **C** Metagenomic abundance of MAGs of *Chrysochromulina* and *Thalassiosira* along the entire spring bloom timeline (epilimnion and hypolimnion). **D** Heatmap of metagenomic abundances of MAGs of *Chrysochromulina* and *Thalassiosira* across several freshwater metagenomic datasets. A scale is shown to the right. *Chrysochromulina* is abbreviated as “Chryso.” in **C** and **D**

The genus *Chrysochromulina* is broadly distributed in marine and freshwater ecosystems [72, 73] and has been documented as an under-ice bloomer in nutrient-poor conditions in habitats influenced by freshwater [74]. These mixotrophic algae play crucial ecological roles in global carbon sequestration and bloom formation and are a rich food source for an extensive range of grazers [75–77]. Average nucleotide identity comparisons between all bins of *Chrysochromulina* suggested the presence of two distinct clusters with 11 (group 1) and 8 bins (group 2) each (Additional file 1: Figure S13). As most of these bins had genome completeness estimates of less than 40%, we collapsed both groups [78] to improve genome recovery and obtained much-improved genome completeness estimates for both groups (51% for group 1 and 44% for group 2) and genome size approached 59 Mb for both groups (group 1, 59.7 Mb and group 2, 59.97 Mb). These genome sizes are comparable to the available genomes of freshwater *Chrysochromulina* isolates (*C. tobinii:* 59 MB, 58% completeness; *C. parva:* 65 MB, 68% completeness) [79, 80]. Comparisons of these recovered bins to the two available *Chrysochromulina* genomes indicated extremely high genomic identities, particularly to group 1 MAGs (Additional file 1: Figure S13). This was also supported by phylogenomic analyses using conserved eukaryotic marker genes (Fig. 4). As mentioned before, a haptophyte bloom was visible after the main phytoplankton bloom (predominantly cryptophytes) on days 21 and 23, accounting for nearly 20% of all rRNA sequences (Fig. 2), which is also in line with a maximum in metagenomic fragment recruitment of the recovered genomes (Fig. 4).

Similarly, for *Thalassiosira* (a centric diatom), we obtained four metagenomic bins that were ca. 50% complete (Additional file 7: Table S6). These MAGs had nearly 100% average nucleotide identity to each other but very low ANI to available *Thalassiosira* genomes (Additional file 1: Figure S13). We merged these MAGs into a consensus genome that was ca. 31 MB in size and estimated to be 63% complete. Genome sizes from cultures of *Thalassiosira* appear highly variable, i.e., *T. pseudonana* (freshwater and brackish) [81] and *T. oceanica* (marine) [82] have quite different genome sizes ranging from 29 to 90 MB, respectively. Phylogenomic analysis using all available genomes and transcriptomes from centric diatoms (*Coscinodiscophyceae*) also placed this MAG within the genus *Thalassiosira* (Fig. 4). This is surprising as *Thalassiosira* are usually considered marine species and *Cyclotella* is understood to be prevalent in freshwater lakes [83]. Our microscopic identification, in sharp contrast to the phylogenomic analyses also potentially identified this organism as a *Cyclotella.* Phylogenomic analyses, however, suggest (Fig. 4) that the genus *Cyclotella* is perhaps polyphyletic. In any case, the organismal genome recovered here, according to genomic analyses appears better placed to be classified as a *Thalassiosira* and not *Cyclotella*.

The freshwater *Thalassiosira* appears to be present only at the beginning of the spring bloom and declines thereafter, in line with 18S rRNA screening (Fig. 2), and its profile is similar in both epi- and hypolimnion. We could also detect the *Chrysochromulina* and *Thalassiosira* MAGs in metagenomes from different locations in Europe and North America (Fig. 4), indicating that the organisms represented by these MAGs are widely distributed across large geographical distances.

Since the discovery of Nucleo-Cytoplasmic Large DNA Viruses (NCLDV, phylum Nucleocytoviricota) [84, 85], giant viruses continue to be isolated from diverse protists, e.g., the kinetoplastid *Bodo saltans* [86], the bicosoecid *Cafeteria roenbergensis *[87], and the marine coccolithophore *Emiliana huxleyi* that forms vast phytoplankton blooms that can be visualized from space [88]. Giant viruses are not only unique in that they have extremely large genomes (in excess of 1 MB), but also that they encode a nearly complete protein translation apparatus hitherto unknown for any other viruses. Metagenomic identification of giant viral genomes from diverse habitats and as endogenous viruses in protist genomes has recently exposed a vast diversity [89–91]. In the marine habitat, it has been recognized that giant viruses infecting *E. huxleyi* are important contributors to bloom collapse [92, 93], supporting aggregation of particles, higher zooplankton grazing, and increased transfer of carbon to deeper water levels [88]. However, abundant giant viruses from freshwater habitats, particularly their dynamics in a high-frequency sampling regime, remain largely unknown. We recovered 3309 contigs from nucleocytoplasmic large DNA viruses infecting microbial eukaryotes using ViralRecall (see the “Methods” section). These contigs ranged from 10 to 266 kb and were dereplicated to 1721 clusters.

Phylogenetic analysis using concatenated markers and protein sequence comparisons with a curated set of 939 reference NCLDV genomes [89–91] revealed that most of these NCLDVs belong to the order Imitervirales (Additional file 8: Table S7, Additional file 1: Figure S14). Additional comparisons to known NCLDV viruses with CheckV [94] suggested similarities to viruses infecting haptophytes (*Chrysochromulina ercinia* virus, 99 contigs; *Phaeocystis globosa* virus 163 contigs) and the chlorophyte* Tetraselmis* (Tetraselmis virus, 41 contigs). In total, tentative host assignment was possible for only 323 NCLDV contigs (Additional file 8: Table S7). This relatively low prediction rate is largely owing to the absence of NCLDV genomes/isolates from dominant spring bloom participants. Fragment recruitment of the NCLDV contigs across the entire time revealed short-lived peaks that suggest a near-continuous turnover in the epilimnion in contrast to deeper waters where their abundances appeared more stable (Additional file 1: Figure S15). The role of viruses in the top-down control of unicellular eukaryotes (the viral shunt) [95, 96] has long been understood to be vital in the release of organic and inorganic nutrients. Additionally, NCLDV viruses have been implicated in the collapse of the vast blooms of the marine haptophyte *Emiliana huxleyi* [54, 97]. Taken together, it is not inconceivable that NCLDVs may have a similarly important role in bloom collapse in the epilimnion.

### Dynamics of plastidic and aplastidic cryptophytes

**Fig. 5:**
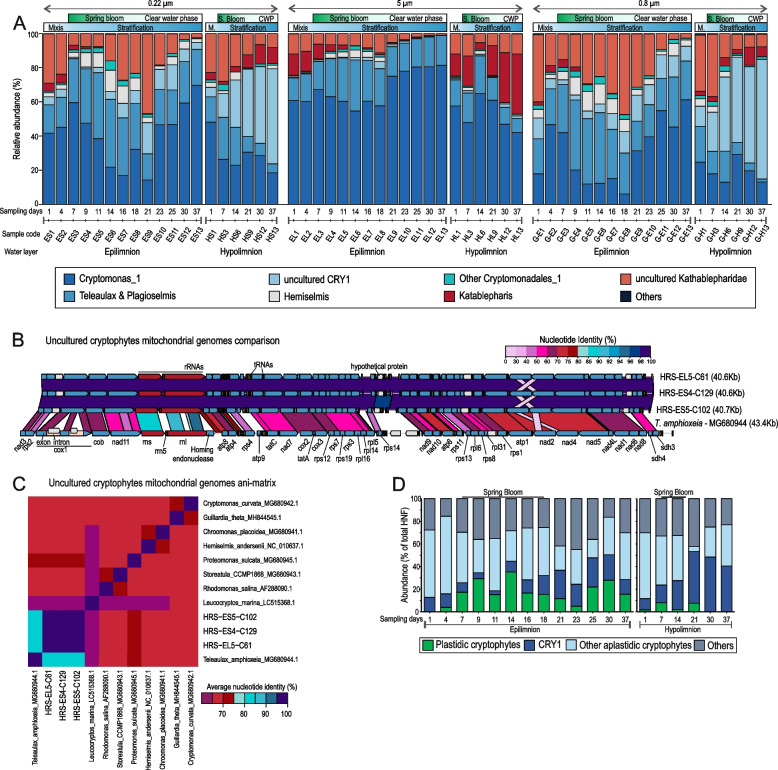
**A** Dynamics of cryptophytes and closely related group Katablepharids during the spring bloom assessed by 18S rRNA gene read sequences. Results are shown as the percentage of total cryptophyte reads recovered. **B** Genomic comparison (nucleotide-nucleotide) of three complete uncultured cryptophytes mitochondrial genomes recovered here with the only available reference mitochondrial genome for *Teleaulax amphioxeia*. A scale is shown at the top right. **C** Average nucleotide identity (ANI) of uncultured cryptophyte mitochondrial genomes in comparison to all known cryptophyte mitochondrial genomes. **D** CARD-FISH abundance estimates for plastidic and aplastidic cryptophytes are shown as % of total eukaryotes. Sampling days are indicated along the *X*-axis

As cryptophytes are major players in the spring bloom (Fig. 1), we attempted to assess the abundance of different cryptophyte groups. As noted before, there appear to be two peaks of cryptophytes highlighted by microscopic observations and rRNA gene sequence abundance information (Figs. 1 and 2). However, microscopic observations suggest the first peak being larger than the second (Fig. 1), while rRNA gene sequence abundance peaks suggest two nearly equal maxima (Fig. 2). Additionally, most cryptophyte 18S rRNA gene reads were associated with *Cryptomonas* or *Teleaulax*/*Plagioselmis* (Fig. 5). *Teleaulax* has been reported before in arctic under-ice spring blooms [98] and in annual blooms in the Columbia estuary [23]. Consistent with this, we also recovered three complete and circular mitochondrial genomes that appeared to originate from a *Teleaulax-*related microbe. These genomes are considerably distinct from the published *Teleaulax* mitochondrial genome [99] (ca. 80% nucleotide identity) but, nevertheless, syntenic to the latter apart from the lack of introns from the Cox1 gene in the assembled mitochondrial genomes (Fig. 5). A comparison of these mitochondrial genomes to all other available mitochondrial genomes also indicated *Teleaulax* as the closest neighbor (Fig. 5). Given the relatively low nucleotide identity, it is more likely that they originate from an uncultured cryptophyte.

Apart from *Cryptomonas* and *Teleaulax-like* sequences, which both belong to the order Cryptomonadales, the as-yet uncultured aplastidic CRY1 group (which does not belong to Cryptomonadales) appears also to be dominant (Fig. 5). The uncultured CRY1 group has been described as among the major bacterivorous flagellates in freshwaters [100, 101] (Fig. 5). While CRY1 has been shown to be potentially a major bacterivore in the epilimnion [31], where a peak is also evident (Fig. 5), its abundances in deeper water strata have remained less examined. With increasing stratification, the abundance of CRY1 rises gradually in the hypolimnion (Fig. 5). However, the abundance of the uncultured CRY1 alone does not explain the Cryptomonadales associated 18S rRNA gene abundances in the epilimnion (Fig. 1). Such discrepancies between microscopic counts and sequencing have been described before, specifically for cryptophytes, including the CRY1 lineage that may be under-represented [102] and other protists, e.g., diplonemids that may be over-represented [103]. This inconsistency between sequence-based abundances and microscopic counts for cryptophytes may either be explained by non-photosynthetic or aplastidic cryptophytes within the order Cryptomonadales that are closely related to *Cryptomonas* (e.g., *Chilomonas*). This inference is supported by the observation that another known aplastidic lineage (apart from CRY1) represented by *Goniomonas *[104] was practically absent in this time period and most 18S rRNA gene reads could clearly be classified as Cryptomonadales. Hence, we suspect that these aplastidic cryptophytes (non-CRY1) are likely related to photosynthetic cryptophytes and are responsible for the second peak, while the first peak is primarily caused by photosynthetic cryptophytes as is also evidenced by elevated chlorophyll-*a* concentrations (Fig. 1).

To ascertain the prevalence of non-photosynthetic cryptophytes in this time period, we performed CARD-FISH analyses on the entire timeline in both the epi- and hypolimnion for cryptophytes using the general cryptophyte probe CryptoB (targeting all plastidic and aplastidic cryptophytes) and a specific probe for the aplastidic CRY1 lineage [100]. In addition, we also enumerated plastidic (photosynthetic, CryptoB positive) and aplastidic cryptophytes (non-photosynthetic, CryptoB positive). CARD-FISH results revealed an even higher abundance of aplastidic cryptophytes than hinted at by sequence data (Fig. 5).

The aplastidic CRY1 lineage is well-represented pre-bloom (ca. 10%) and increases gradually, reaching an abundance of 20–30% of total eukaryotes in the epilimnion. More pronounced increases for CRY1 were observed in the hypolimnion, in line with 18S rRNA sequences, where they constituted ca. 40–50% of total eukaryotes (Fig. 5). Plastidic cryptophytes, on the other hand, are practically absent before the bloom, start to increase only around day 4 and reach max. 40% of total eukaryotes at day 14. In contrast, aplastidic cryptophytes are already the most prominent HNF, accounting for ca. 70% of total eukaryotes at day 1, and were highly abundant throughout the entire spring bloom period. Moreover, during plastidic cryptophytes’ bloom, only small abundance reductions are visible for aplastidic cryptophytes, which nearly always constitute the most dominant eukaryotes. This also suggests that the population dynamics of aplastidic cryptophytes are relatively independent of plastidic cryptophytes (or other algae) as they are bacterivores [100, 101]. The identity of these aplastidic cryptophytes remains a mystery as they are indistinguishable from their plastidic counterparts by 18S rRNA gene sequences. Their dominance during the algal spring bloom, which has so far been perceived as a primarily phototrophic event, is unexpected and might cause a paradigm shift in understanding the role of heterotrophic nanoflagellates during the spring bloom.

### Concluding remarks

**Fig. 6:**
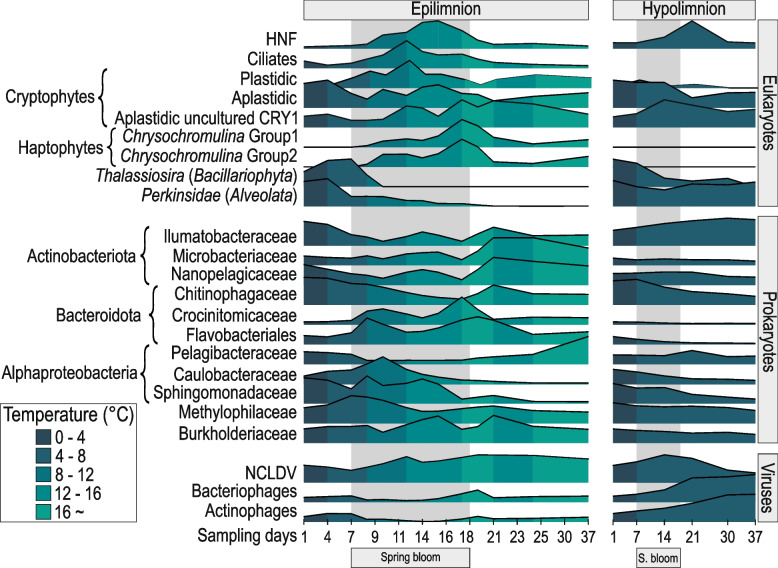
Schematic overview of the spring bloom in Římov reservoir. Both Epilimnion and Hypolimnion are shown. Abundances (minimum to maximum, scaled from 0 to 1) from multiple methods are shown (microscopy, CARD-FISH, 18S rRNA abundances, and cumulative MAG abundances from all three filter sizes). Microscopy: HNF and Ciliates, CARD-FISH: Plastidic, Aplastidic, and uncultured CRY1 cryptophytes; 18S rRNA abundances: Perkinsidae; MAG abundances: *Chrysochromulina* and *Thalassiosira*; all prokaryotes and viruses. All prokaryotic MAGs and viral/NCLDV contigs were dereplicated. A temperature scale is shown at the bottom left, and the spring bloom period is highlighted in a gray box

The multiple data sources at hand, the combination of different methods, and the identification of major participants allowed us to reconstruct the sequence of events that play out in the theater of the spring bloom in far greater detail than was possible before (Fig. 6). The integration of microscopic and metagenomic data facilitated the discovery of the most abundant players in the spring bloom of the Římov reservoir. We identified multiple successional patterns across diverse and abundant prokaryotic groups. While Caulobacteraceae and some genera of Crocinitomicaceae and Flavobacteriaceae seem to be bloom specialists, ‘*Ca.* Fonsibacter’ and ‘*Ca.* Nanopelagicus’ seem to avoid the phytoplankton spring bloom (Figs. 5 and 6). Other genera like *Limnohabitans* or ‘*Ca.* Planktophila’ show a more complex pattern with multiple (sometimes even closely related) populations with striking differences in abundance. The high-resolution sampling data also allowed us to compute mortality-corrected doubling times for several abundant planktonic taxa. For some bacteria, their transitions between the epi-and hypolimnion could be linked to temperature, but additional evidence linking metabolically active genes was not generated in this work. Also, in the hypolimnion, some phages reach very high abundances, indicative of a massive phage infection (Fig. 6) in the clear water phase.

The dominance of aplastidic cryptophytes in the spring bloom and their persistent abundances suggest decoupling from their plastidic counterparts and also suggest that there are abundant aplastidic ‘cryptic’ cryptophytes that comprise a large fraction of unicellular eukaryotes and whose identities are as yet unknown. Parasites and viruses have been considered as potential biological drivers of algal bloom collapse additionally to zooplankton grazing [2]. However, Perkinsozoa appears far more abundant before the bloom and in the hypolimnion, whereas NCLDV can be found at high abundances in both epi- and hypolimnion suggesting that top-down control by NCLDV, in tandem with bottom-up controls of nutrient limitations may be more relevant for bloom collapse than perkinsids. Perkinsids on the other hand show profile abundances similar to aplastidic cryptophytes (including CRY1) (Fig. 6) and may be parasitizing aplastidic cryptophytes as shown before for *Cryptophagus* infecting *Chilomonas* [53].

Applying high-temporal resolution sampling using multiple methods to a single site increased our ability to discern several biological patterns. However, many aspects remain unclear, e.g., specific interactions between eukaryotes and prokaryotes, and even between eukaryotes themselves, the nature of metabolites supplied by the phytoplankton bloom to the copiotrophic bloom specialists, and specific genomic adaptations that distribute prokaryotes to specific niches or enable them to cooperate, e.g., Black Queen hypothesis [105]. Moreover, the adaptations of viruses (both phages and NCLDV) and the identity of their hosts remain mostly unknown. The annual spring bloom, with its dynamic nature and compressed view of planktonic succession, still withholds many hidden ecological mysteries that will only be revealed by future work.

## Material and methods

### Study site and sampling

This study was conducted in the canyon-shaped meso-eutrophic Římov Reservoir (Czech Republic; 48° 50′ 56′′ N, 14° 29′ 26′′ E; 470 m a.s.l.; area 2.06 km^2^; volume 34.5 × 10^6^ m^3^; length 13.5 km; max. depth 43 m; mean depth 16.5 m; mean retention time 77 days; dimictic) [106]. The reservoir was built as a drinking water reservoir by impounding River Malše, the main reservoir tributary, accounting for 90% of the water inflow. Římov Reservoir is also part of the Czech Long-Term Ecological Research network [107]. Water samples were collected from April 3 to May 9, 2018, 3 times a week except for the last week of the study period covered with a weekly sampling. Zooplankton composition was analyzed at weekly intervals.

### Vertical profile of different physicochemical factors

A multi-wavelength submersible fluorescence probe (FluoroProbe, bbe-Moldaenke, Kiel, Germany) was employed to measure chlorophyll-*a* concentrations in a discrete vertical profile at ~ 0.2 m intervals down to the bottom. According to the specific fluorescence spectra of distinct phytoplankton groups, the probe permits differentiation of cyanobacteria, chromophytes + dinoflagellates (a mixed group with diatoms frequently most important), cryptophytes, and Chlorophyta in mixed natural populations [108]. A submersible multiparametric probe (YSI EXO 2, Yellow Springs Instruments, Yellow Springs, USA) was deployed to measure detailed vertical profiles of temperature, pH, oxygen concentrations, and conductivity (Additional file 1: Figure S16).

Fixed depths of 0.5 m and 30 m were chosen for further analyses and measurements. Samples (20 l) were collected above the deepest point at the dam using a Friedinger sampler, transported to the laboratory within 30 min, and split into subsamples for microbial enumeration, CARD-FISH, metagenomic filtration, and chemical analyses. Samples for dissolved silica (DSi) and dissolved reactive phosphorus (DRP) assessments were filtered through filters of 0.4-μm porosity (Macherey Nagel GF-5, Macherey Nagel, Düren, Germany) in the laboratory. DSi and SRP were determined spectrophotometrically according to [109, 110], respectively. Total phosphorus (TP) and concentrations of NO_3_^−^–N were analyzed as described before [111, 112].

Phytoplankton samples were preserved with Lugol’s solution and stored in the dark. Species were enumerated employing the Utermöhl method and an inverted microscope (Olympus IX 71) [113]. The mean algal cell dimensions were obtained for biovolume calculation using the approximation of cell morphology to regular geometric shapes[114].

### Bacterial, protistan, and VLP counts; protistan bacterivory rate; and CARD-FISH analyses

Subsamples of 20 ml were fixed with formaldehyde (2% final concentration), stored at 4 °C, and processed within 24 h after sampling. Total bacterial abundance was quantified via flow cytometry in samples stained with the fluorochrome SYBRgreen (Molecular Probes) using a CytoFLEX S flow cytometer (Beckman Coulter), equipped with a blue laser and bandpass filters 525/40 and 690/50. Cell sizing (> 300 cells per sample) based on measuring cell width and length parameters in 4′,6-diamidino-2-phenylindole (DAPI)-stained preparations [115] was conducted by using a semiautomatic image analysis system (NIS-Elements 3.0, Laboratory Imaging, Prague). Bacterial biomass was calculated based on volumetric formulas and volume-to-carbon conversion factors [115]. Details of the groups examined are provided in Additional file 2: Table S1.

Duplicates of Lugol–formaldehyde–thiosulfate fixed subsamples [31] of 5–10 ml (HNF) and 15–45 ml (ciliates) were stained with DAPI, filtered onto a black 1.0-μm pore-size filter (Osmonic, Livermore, CA), and HNF and ciliate abundances were determined via epifluorescence microscopy as described elsewhere [6]. Protistan bacterivory was estimated using fluorescently labeled bacteria (FLB) [116] prepared from a mixture of two *Limnohabitans* strains and one strain of the PnecC lineage of *Polynucleobacter* as detailed before [117]. The sizes of FLB reassembled the typical size class distribution of the reservoir bacterioplankton with a mean cell size of around 0.065 μm^3^. HNF and ciliate FLB uptake rates were determined in short-term FLB direct-uptake experiments by inspecting protist cells [118], with ciliates being determined to species, genus, or morphotype level (for details, see [24, 117]). To estimate total protistan grazing, we multiplied the average uptake rates of HNF and ciliates by their in situ abundance.

To quantify virus-like particles, 1 ml from each water sample was fixed with glutaraldehyde (1% final concentration) for 10 min, shock-frozen in liquid nitrogen, and stored at − 80 °C. Enumerations of VLP were done using an Influx V-GS cell sorter (Becton Dickinson) as previously described [119].

### CARD-FISH for prokaryotes

Formaldehyde-fixed subsamples (5 ml) for CARD-FISH were filtered onto 0.2-μm pore-sized polycarbonate filters (Millipore, Merck, Darmstadt, DE) and stored at − 20 °C. CARD-FISH was conducted as previously described [120] using fluorescein-labeled tyramides and the following HRP-labeled oligonucleotide probes: Ac1-852 [121] targeting the order *‘Ca*. Nanopelagicales’ (acI Actinobacteria), Npel-2669 [32] targeting the genus *‘Ca.* Nanopelagicus’ (acI-B1), MET1217 [122] targeting the family Methylophilaceae, LD28-1017 [123] targeting the genus ‘*Ca*. Methylopumilus’, and LD12-115 [124] targeting the genus *‘Ca.* Fonsibacter’ (LD12). Filters were counterstained with DAPI and analyzed by epifluorescence microscopy (Zeiss Imager.Z2, Carl Zeiss, Oberkochen, DE) with a colibri LED light system. Images were recorded with an Axiocam 506 (Carl Zeiss, Oberkochen, DE) and analyzed with the software ACME-tool (www.technobiology.ch) as outlined before [16].

### CARD-FISH for eukaryotes

Water samples fixed with Lugol-Formaldehyde-Thiosulfate [125] were filtered on polycarbonate filters (pore size 0.8 μm, Millipore) within 24 h of fixation, and filters were stored at − 20 °C until further processing. Two oligonucleotide probes were used in the present study targeting all cryptophytes (CryptoB) [126] and their monophyletic CRY1 lineage (Cry1-652) [100] with the hybridization conditions described before [127]. CARD-FISH was performed with tyramides [118] labeled with fluorescein according to the method described recently [31]. CARD-FISH preparations were analyzed and enumerated with an epifluorescence microscope (Olympus BX 53) at 1000 × magnification under blue/UV excitation. Total microbial eukaryotes were counted simultaneously from the same preparations with the DAPI counter-stain under UV excitation. Images of CARD-FISH targeted cells were captured and processed using a semi-automatic image analysis system (NIS-Elements 3.0, Laboratory Imaging, Prague, Czech Republic).

### Zooplankton abundance and community composition

Crustaceans were sampled weekly by vertical hauls using an Apstein plankton net (200 μm mesh size). Two vertical hauls were taken from 5 m to the surface (755 l in total), representing the depth of epilimnion, where usually 90% of the crustaceans from the whole water column are accumulated [128]. Rotifers were sampled from the uppermost 5 m of the water column using a plastic tube of the appropriate length. Forty liters of the sampled water were subsequently concentrated using a 30-μm net. Zooplankton samples were preserved in 4% formaldehyde, and the abundance of the main zooplankton species was determined microscopically [129]. The final counts were normalized by the total amount of filtered water (Additional file 1: Figure S17).

### Metagenomic filtration and DNA extraction

In total, fifty-seven DNA samples were collected from epilimnion (0.5 m, *n* = 39) and hypolimnion (30 m, *n* = 18) using three different filter-pore sizes (5 μm, 0.8 μm, and 0.22 μm) and two types of filtration procedures (positive pressure and gravity filtration). More details about the samples are available in Additional file 2, Table S1. Two of these metagenomic datasets have been published previously [20]. The rest were generated in this study.

### Positive pressure filtration

Water samples (ca. 20 L) from both depths were sequentially filtered through a 20-μm mesh plankton net to remove larger organisms, followed by 5-μm and 0.22-μm polycarbonate membrane filters using a peristaltic pump until filters were clogged. A 5-μm and 0.22-μm fractions were collected and stored at − 80 °C until DNA extraction.

### Gravity filtration

Water samples (ca. 20 L) from both depths were gravity filtered through a 20-μm-mesh plankton net, followed by 5-μm and 0.8-μm filters, as described before [100]. DNA was extracted only from the 0.8-μm filters.

All filters were cut into small pieces (≅ 3–5 mm) using sterile scissors and processed for DNA extraction using the ZR Soil Microbe DNA MiniPrep kit (Zymo Research, Irvine, CA, USA), according to the manufacturer’s instructions.

### Preprocessing and assembly of metagenomic datasets

DNA samples were sequenced using Illumina Novaseq 6000 platform (Novogene, Hong Kong, China). Low-quality bases, reads, and adaptors were trimmed using the bbmap package (https://sourceforge.net/projects/bbmap/). Raw reads were interleaved, and quality trimmed by reformat.sh, followed by bbduk.sh (Phred score = 18). Additionally, bbduk.sh was used to remove any adapter or PhiX and p-Fosil2 contamination. A final check using bbmerge.sh was done to ensure the quality threshold for assembly of the contigs. MEGAHIT (v1.1.5) was used with default settings to assemble the preprocessed reads (k-mer sizes: 49,69,89,109,129,149) [130].

### 16S and 18S rRNA gene abundance-based taxonomic classification

Each metagenome was randomly subsampled (20 million reads) and compared to SILVA v138 database [131] to detect candidates of 16S and 18S rRNA gene reads (*e* value 1e^−5^) using MMSeqs2 [132]. The putative rRNA reads were scanned with ssu-align (http://eddylab.org/software/ssu-align/) to identify 16S rRNA and 18S rRNA sequences. Lastly, the taxonomy classification was attributed using blastn [133] against the SILVA database v138. For 18S rRNA classification, sequences originating from organisms known to have thousands of rRNA operons, e.g., Dinoflagellates, Ciliophora, and those from multicellular organisms, i.e., Metazoa, were ignored. For both 18S and 16S rRNA, sequences originating from Chloroplasts, Mitochondria, and Nucleomorphs of Cryptophyceae were excluded from the analysis. The sequences were classified based on 95% identity to the databases.

### Recovery of prokaryotic metagenomic assembled genomes

Preprocessed metagenomic datasets were mapped using bbwrap.sh (kfilter = 31 subfilter = 15 maxindel = 80) against the assembled contigs (≥ 3 kb). The contig abundance files were obtained using jgi_summarize_bam_contig_depths [134] and used for the binning process with MetaBAT2 with default parameters [134]. Contigs suspected to be of viral origin by scanning with VIBRANT [135] or ViralRecall [136] were excluded. An additional cleaning step, considering the homogeneity of the contigs in each bin, was performed in order to assure high-quality bins: Open reading frames from each contig inside the bins were predicted using PRODIGAL v2.6.3 [137], and taxonomy of each gene was assigned using MMseqs2 [132] with Genome Taxonomy DataBase (version r95) (GTDB) [138]. Contigs with more than 30% of genes with no hits or hits to eukaryotes or viruses as well as contigs in which the taxonomy disagreed with the consensus class were removed.

The taxonomy of each bin was compared to the taxonomy of 16S and 23S rRNA gene sequences within the bin. Briefly, rRNA sequences were identified using barrnap with default parameters (https://github.com/tseemann/barrnap), extracted, and compared to the SILVA v138 database [131]. Simultaneously, the taxonomy of the whole bin was assigned using the GTDB-Tk [139] toolkit based on the GTDB taxonomy (version r95). Contigs in which 16S or 23S rRNA taxonomic classification did not match with GTDB taxonomy were removed. The resulting bins were evaluated with CheckM v1.0.18 [140], and only those with > 40% completeness and < 5% contamination were retained for further analysis (*n* = 2214). These bins were further dereplicated using dRep (-comp 40 -con 5) [141], resulting in 855 representative prokaryotic bins.

### Recovery of phage genomes, dereplication, and host prediction

In order to obtain complete phage genomes, assembled circular contigs > 10 kb (*n* = 1225) were used for the downstream analysis as described before [20, 58]. The selected contigs were scanned using VIBRANT—default settings [135]. Lastly, an additional step of manual curation using the NCBI Batch CDD server [142] was performed to enhance the accuracy of phage recognition. We recovered 679 bonafide complete phage genomes with this protocol.

We clustered phages by an all-vs-all BLASTN approach, retaining only significant matches (1e − 3). If, in comparison, two phages had genome coverage of > 95% and > 95% nucleotide identity, they were considered part of a cluster. Next, we used a single linkage to merge all potential clusters. Finally, we obtained 175 distinct phage genome clusters. Multiple approaches were used to link the recovered phages to a putative host; for example, we searched for photosystem genes in phage genomes that are a hallmark for a cyanobacterial host [143]. Similarly, the *whiB* transcriptional regulator is a marker gene for phages infecting Actinobacteria. and *whiB* for Actinobacteria [58]. Additionally, phages frequently insert in tRNA loci (termed *attB*) in the host genome, and the presence of such a site in the phage genome may imply a specific association [144]. We used BLASTN [133] for examining such identities between putative attB sites and phage genomes as described before (alignment length ≥ 30 bp, ≥ 97% nucleotide identity, ≥ 97% query coverage, ≤ 1e − 5) [144]. We also used CRISPR spacers (detected using minced, available from https://github.com/ctSkennerton/minced) from the recovered microbial genomes to query phage genomes using BLASTN with stringent cutoffs (alignment length ≥ 30 bp, ≥ 97% nucleotide identity, ≥ 97% query coverage, ≤ 1e − 5). Finally, we made direct comparisons of phage genomes to host genomes using BLASTN to identify shared nucleotide sequences (alignment length ≥ 30 bp, ≥ 97% nucleotide identity, ≥ 97% query coverage, ≤ 1e − 5) [145].

### Recovery of NCLDV genomes and phylogenomics

All contigs longer than 10 kb were scanned with ViralRecall [136] to identify signatures of putative nucleocytoplasmic large DNA viruses (NCLDVs). For capturing bonafide NCLDVs, we applied a strict threshold of at least five viral hits, at least one marker gene, and a score ≥ 1. Following this, we manually curated these contigs by examining hits to multiple databases, some contigs were removed. Finally, 3309 were finally retained as bonafide NCLDV contigs. (results in Additional file 8, Table S7).

A collection of previously published NCLDV genomes (*n* = 1447) was generated from (1) annotated genomes in GenBank, (2) giant virus MAGs published by Schulz et al. [91], and (3) a highly curated and taxonomically coherent collection of giant viruses compiled by Aylward et al. [90]. All viruses were dereplicated together with dRep [141] (default parameters; completeness and contamination data not used for scoring) resulting in 1245 unique genomes. Dereplication was performed in the same manner for NCLDV contigs retrieved in this study (*n* = 3309) resulting in *n* = 1721 representative sequences. Information regarding viral clusters and the number of members within each cluster are included in Additional file 8: Table S7. All dereplicated genomes (references and the ones retrieved in this study) were scanned with ncldv_markersearch [146] for the presence of 7 giant virus marker genes (GVOGm) previously selected due to their strong phylogenetic signal [90]. We discarded all instances with less than 2 identifiable markers resulting in *n* = 1240 reference genomes and *n* = 153 viral contigs from this study. Protein markers retrieved from all genomes were individually aligned using Clustal Omega [147], trimmed with BMGE (-t AA -g 0.5 -b 3 -m BLOSUM30 -g 0.5) [148] and concatenated. An initial maximum likelihood (ML) phylogenomic tree was constructed using IQ-TREE v2.1.3 [149] (1000 iterations for ultrafast bootstrapping [150] and SH testing, respectively; best model chosen automatically by ModelFinder [151]: LG + F + I + G4. Upon inspection of the initial tree followed by manual curation (removal of references from overrepresented groups) a final phylogenomic tree was generated including *n* = 940 reference genomes and without changes to our own set (Additional file 8: Table S7). The final tree was annotated to include labels containing the original genome/contig name and taxonomic classification derived from the study of Aylward et al. [90]. All data associated with the published phylogenomic tree is made available in FigShare (https://figshare.com/s/5150884f3fbb534c0302).

### Recovery of organellar genomes

Circular contigs (> 30 bp overlap at the end) were compared using MMSeqs2 to a database containing all GTDB proteins (r95) and all UniProt eukaryotic and viral proteins. Additionally, we used metaeuk [152] in easy-predict mode vs the UniProtKB database. The results were manually examined, and those that appeared to be of mitochondrial or chloroplast origin were segregated. Details of organellar genomes recovered in this work are provided in Additional file 9: Table S8.

### Recovery of eukaryotic metagenome-assembled genomes

All bins that could not be classified as prokaryotic by GTDB-Tk [139] and CheckM [140] were input to BUSCO [153] in –auto-lineage mode to identify candidate eukaryotic MAGs. Once a candidate MAG was identified, we used metaeuk [152] in easy-predict mode vs the UniProtKB [154] and MMETSP [155] databases to identify the taxonomic origin of contigs. Contigs that did not conform to the majority taxonomic consensus within a MAG were discarded. After cleaning the bin, we estimated genome completeness using BUSCO [153] again with specific lineage datasets wherever possible.

### Prokaryotic and eukaryotic phylogenomic analyses

Maximum-likelihood (ML) phylogenomic trees were constructed for major bacterial groups using IQ-TREE2 (v.2.1.2) with 1000 iterations of ultrafast bootstrapping [150] and SH testing [149]. The best fitting evolutionary models were automatically determined for each group by activating the ModelFinder [151] option of IQTREE. Reference genomes and outgroups were selected from a curated collection of ~ 100,000 uniformly classified genomes (using gtdb-tk, r95) which were scanned with hmmsearch together with the ones recovered in this study for the presence of 120 conserved protein HMM markers [138]. Amino-acid sequences for each of the 120 makers were aligned using PASTA [156] and trimmed by BMGE (parameters: -t AA -g 0.5 -b 3 -m BLOSUM30) [148]. Individually trimmed alignments were concatenated using catfasta2phyml.pl (https://github.com/nylander/catfasta2phyml) and finally used as input for IQTREE. All details about selected models and alignment statistics are available in Additional file 10: Table S9.

BUSCO [153] was used to identify single-copy genes in recovered eukaryotic MAGs, available transcriptomes, or genomes. For *Thalassiosira* and *Chrysochromulina* phylogenomic trees, 71 and 68 conserved single copy markers were used, respectively. Only those genomes/transcriptomes with at least 70% of these markers were used. Alignments, trimming, concatenation of aligned markers, and construction of phylogenomic trees were performed as described above for prokaryotic genomes.

### Fragment recruitment, estimation of replication, and duplication times

From each metagenome, 20 million quality-filtered reads were mapped against the recovered genomes using RazerS 3 (–no-gaps, –max-hits 1,000,000) [157]. All alignments were filtered to at least 50 bp and 95% nucleotide sequence identity. The obtained number of hits were used to compute coverage per Gbp values, offering normalized abundances comparable for different MAGs and metagenomes. Accession numbers of the publicly available metagenomic datasets used for the recruitment shown in Fig. 4D are listed in Additional file 11: Table S10.

We used GRiD for estimating the rate of bacterial replication (GRiD multiplex module with default options) [158]. According to the recommendation, we used only forward reads from each metagenome.

For computing doubling times using gRodon, only dereplicated MAGs were used. Additionally, only those MAGs that had a minimum of 15 ribosomal genes and 500 predicted genes were retained. The doubling times for each genome were estimated with the default parameters using gRodon with the appropriate sample temperature as recommended [69]. The same MAGs (considered high-quality MAGs for doubling time estimations) were further used for computing doubling times using metagenomic abundances.

Estimation of doubling times as performed here (both using CARD-FISH or metagenomic abundances) was done according to the following assumptions: Firstly, estimates of average mortality by grazing (24%) and by viruses (31%) were obtained from prior observations from a relatively large sample of 19 lakes [68]. Taken together, a total of 55% mortality was assumed as a combined effect. Additionally, for all groups examined, only those adjacent time points that showed a change of at least 25% were considered to identify the fastest doubling times which may seem at odds with the constant mortality assumption but can also be considered as ignoring the initial lag phase during exponential growth [4]. As there is usually a strong dependence of sampling site and season upon these values, not all assumptions may be always equally valid. Hence, the doubling times obtained here must be considered rough estimates. The observed in situ doubling time (in hours) was computed as described before [4] for each pair of adjacent time points that showed at least a 25% increase. Briefly, growth rate *r* = 1/(t1-t0) *ln(N2/N1), where t0 and t1 are initial time points (hours), and N1 and N2 are abundance values at t0 and t1, respectively. The doubling time may be computed as ln (2)/r. The final observed in situ doubling times as shown in Table 1 are expressed as an average of all doubling times. The doubling times in absence of mortality (i.e., estimated doubling time) was obtained by correcting for the mortality estimate (in this case 55%) and averaged as shown in Table 1.

## Supplementary Information

** Additional file 1: Figure S1.** Time course of different features of the spring bloom in the hypolimnion. (A) Chlorophyll a concentrations, temperature, heterotrophic nanoflagellates (HNF), viral like particles (VPL), (B) total phosphorus (TP), NH4-N and dissolved nitrogen (DN), (C) silica, dissolved organic carbon (DOC) and dissolved reactive phosphorus (DRP). **Figure S2.** Heterotrophic nanoflagellates and ciliate counts, bacterial ingestion, grazing rate estimations and turnover of observed ciliates in epilimnion. (A) HNF (10^3^ ml ), Bacteria(10^6^ ml), and ciliate (per ml) abundances, (B) Total grazing rates of heterotrophic nanoflagellates (HNF) and ciliates, expressed as number of ingested bacteria/day, (C) Bacteria ingested by HNF (per hour) and ciliates(per hour). A temperature scale is also shown (D) Counts (per ml) of four ciliates identifiable by morphology. The grey region indicates the spring bloom as identified by chlorophyll content. **Figure S3.** (A) Completeness and contamination estimates for 2214 genomic bins recovered in this study. Three categories of genomic bins were defined based on completeness estimated by CheckM: high quality genomic bins (completeness ≥ 90 %) representing 22.6 % (*n* = 502) of total, medium quality genomic bins (completeness ≥ 70-89 %) amounting to 26.8 % (*n* = 594), and partial genomes (completeness 40-70 %) the remaining 50 % (1118). Bins with contamination > 5 % and completeness < 40 % were not analyzed. The histogram parallel to the x axis shows the percentage distribution of bins according to level of completeness while the one parallel to the y axis indicates the percentage distribution of genomes by contamination. (B) Percent distribution of bins according to tRNA gene copy content. (C) Relationship between gene coding density and estimated genome length. (D) Number of MAGs recovered from each dataset (*n*=57). (E) MAG percentage distribution across phyla. **Figure S4**. Dereplicated bacteriophages genome abundances of 0.22µm filter (Cov/Gb) normalized by Z-score in epi and hypoliminon (*n*=175). The rows are clustered by relative abundance by average linkage (with Spearman Rank correlation method). Inferred host taxonomy is shown at the right wherever applicable. **Figure S5.** Pruned phylogenomic tree of Ca. Fonsibacter (Pelagibacteriaceae) metagenome assembled genomes. Relative abundance of selected MAGs during the spring bloom in the small filter (0.22µm) are shown on the right side. Each row was normalized by Z-score and clustered by average linkage (with Spearman Rank correlation method). Ultrafast bootstrap values are shown at each node. **Figure S6.** Pruned phylogenomic tree of Ca. Planktophila (Actinobacteriota) metagenome assembled genomes. Relative abundance of selected MAGs during the spring bloom in the small filter (0.22µm) are shown on the right side. Each row was normalized by Z-score and clustered by average linkage (with Spearman Rank correlation method). Ultrafast bootstrap values are shown at each node. **Figure S7.** Pruned phylogenomic tree of Limnohabitans (Gammaproteobacteria) metagenome assembled genomes. Relative abundance of selected MAGs during the spring bloom in the small filter (0.22µm) are shown on the right side. Each row was normalized by Z-score and clustered by average linkage (with Spearman Rank correlation method). Ultrafast bootstrap values are shown at each node. **Figure S8.** Dereplicated Alphaproteobacteria (Proteobacteria) MAG abundances of 0.22µm filter (Cov/Gb) normalized by Z-score in epi and hypoliminon (*n*=67). The rows are clustered by taxonomy and relative abundance by average linkage (with Spearman Rank correlation method). **Figure S9.** Dereplicated Bacteroidota MAG abundances of 0.22µm filter (Cov/Gb) normalized by Z-score in epi and hypoliminon (*n*=238). The rows are clustered by taxonomy and relative abundance by average linkage (with Spearman Rank correlation method). **Figure S10.** Dereplicated Gammaproteobacteria (Proteobacteria) MAG abundances of 0.22µm filter (Cov/Gb) normalized by Z-score in epi and hypoliminon (*n*=198). The rows are clustered by taxonomy and relative abundance by average linkage (with Spearman Rank correlation method). **Figure S11.** Dereplicated Planctomycetota and Chloroflexota MAG abundances of 0.22µm filter (Cov/Gb) normalized by Z-score in epi and hypoliminon (*n*=28 and 7,respectively). The rows are clustered by taxonomy and relative abundance by average linkage (with Spearman Rank correlation method). **Figure S12.** Dereplicated Verrucomicrobiota MAG abundances of 0.22µm filter (Cov/Gb) normalized by Z-score in epi and hypoliminon (*n*=52). The rows are clustered by taxonomy and relative abundance by average linkage (with Spearman Rank correlation method). **Figure S13.** Average Nucleotide Identity (ANI) matrix of (A) Chrysochromulina bins, (B) Thalassiosira bins. The color key and a histogram of frequency of identity is shown on the left side. Black squares inside the matrices highlight the groups formed on each matrix. **Figure S14.** Phylogenomic tree of Nucleocytoviricota based on 7 conserved protein markers. Clades were collapsed at Family level while colored rectangles highlight affiliations to different Orders. Family identifiers are provided on the right of each collapsed clade (triangles) followed by square brackets containing the total number of members within the clade (references + genomes from this study) and by the number of dereplicated genomes recovered here from Epilimnion (E) or Hypolimnion (H) if they are present at all. Ultrafast bootstrap values (UFB) are defined by full (90-100) and empty (80-89) circles. UFB values below 80 are not represented. The root was defined between classes Pokkesviricetes and Megaviricetes as in a previous study [90]. **Figure S15.** Dereplicated NCLDV contigs cumulative abundances of 0.22, 0.8 and 5 µm filters (Cov/Gb) normalized by Z-score in epi and hypoliminon (*n*=1721). The rows are clustered relative abundance by average linkage (with Spearman Rank correlation method). **Figure S16.** Limnological parameters in Římov reservoir along the spring bloom (03 April ~ 09 May 2018). **Figure S17.** Total counts of zooplankton during the spring bloom. Rotifera (top) and Crustacea (bottom) abundance. For more details of the sampling scheme please check material and methods. Gray square shown in the background mark the Spring bloom during the time period.

** Additional file 2: Table S1.** Microscopy data - Phytoplankton counts.

** Additional file 3: Table S2.** General statistics and information about the bacterial MAGs.

** Additional file 4: Table S3**. MAGs containing rhodopsins.

** Additional file 5: Table S4.** Bacteriophage general information.

** Additional file 6:Table S5.** FISH counts in epilimnion and hypolimnion.

** Additional file 7: Table S6.** Eukaryotic MAGs information.

** Additional file 8: Table S7.** NCLDV abundances and general information.

** Additional file 9: Table S8.** Details of organellar genomes recovered.

** Additional file 10:Table S9.** Characteristics of phylogenomic trees constructed using IQTREE2.

** Additional file 11: Table S10**. Public datasets used in Fig. 4, pane D.

## Data Availability

All sequence data generated in this work has been deposited to EBI ENA and is available with the bioproject identifier PRJEB52406. Additionally, bacterial and eukaryotic metagenome-assembled genomes, complete phage genomes, NCLDV contigs, alignment files, and trees are also available in figshare (https://figshare.com/s/5150884f3fbb534c0302).
