# Supplementary material for: High-resolution metagenomic reconstruction of the freshwater spring bloom
Source: Microbiome. 2023 Jan 26;11:15. doi: 10.1186/s40168-022-01451-4 (PMC9878933; doi:10.1186/s40168-022-01451-4)
Supplement: Supplementary file 2 — Additional file 1: Figure S1. Time course of different features of the spring bloom in the hypolimnion. (A) Chlorophyll a concentrations, temperature, heterotrophic nanoflagellates (HNF), viral like particles (VPL), (B) total phosphorus (TP), NH4-N and dissolved nitrogen (DN), (C) silica, dissolved organic carbon (DOC) and dissolved reactive phosphorus (DRP). Figure S2. Heterotrophic nanoflagellates and ciliate counts, bacterial ingestion, grazing rate estimations and turnover of observed ciliates in epilimnion. (A) HNF (103 ml ), Bacteria(106 ml), and ciliate (per ml) abundances, (B) Total grazing rates of heterotrophic nanoflagellates (HNF) and ciliates, expressed as number of ingested bacteria/day, (C) Bacteria ingested by HNF (per hour) and ciliates(per hour). A temperature scale is also shown (D) Counts (per ml) of four ciliates identifiable by morphology. The grey region indicates the spring bloom as identified by chlorophyll content. Figure S3. (A) Completeness and contamination estimates for 2214 genomic bins recovered in this study. Three categories of genomic bins were defined based on completeness estimated by CheckM: high quality genomic bins (completeness ≥ 90 %) representing 22.6 % (n = 502) of total, medium quality genomic bins (completeness ≥ 70-89 %) amounting to 26.8 % (n = 594), and partial genomes (completeness 40-70 %) the remaining 50 % (1118). Bins with contamination > 5 % and completeness < 40 % were not analyzed. The histogram parallel to the x axis shows the percentage distribution of bins according to level of completeness while the one parallel to the y axis indicates the percentage distribution of genomes by contamination. (B) Percent distribution of bins according to tRNA gene copy content. (C) Relationship between gene coding density and estimated genome length. (D) Number of MAGs recovered from each dataset (n=57). (E) MAG percentage distribution across phyla. Figure S4. Dereplicated bacteriophages genome abundances of 0.22µm filter ( [file 40168_2022_1451_MOESM1_ESM.pdf]

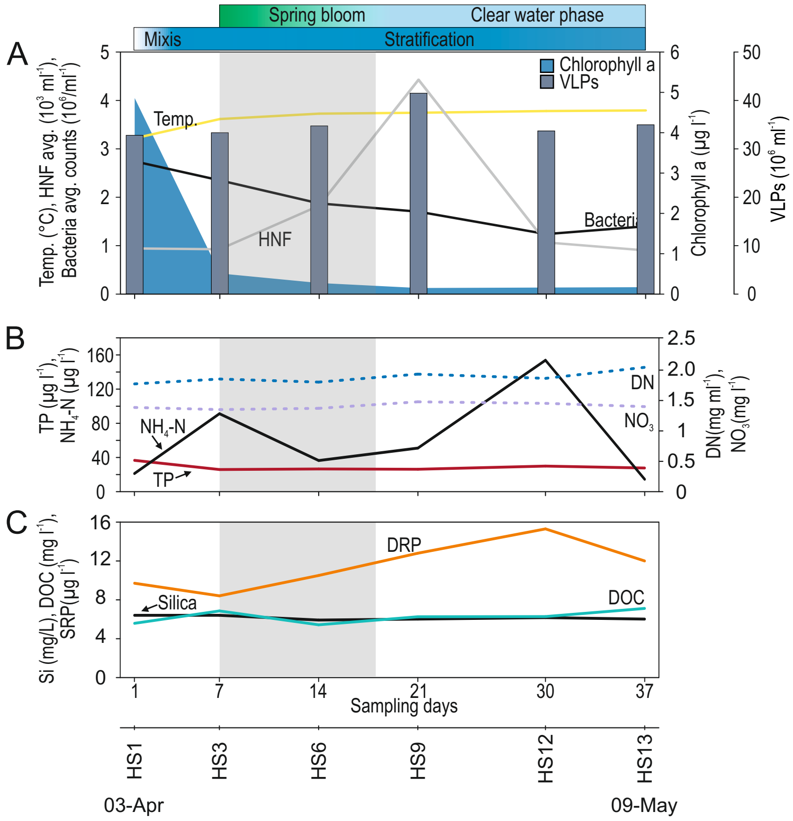

Figure S1. Time course of different features of the spring bloom in the hypolimnion. (A) Chlorophyll a concentrations, temperature, heterotrophic nanoflagellates (HNF), viral like particles (VPL), (B) total phosphorus (TP), NH4-N and dissolved nitrogen (DN), (C) silica, dissolved organic carbon (DOC) and dissolved reactive phosphorus (DRP).

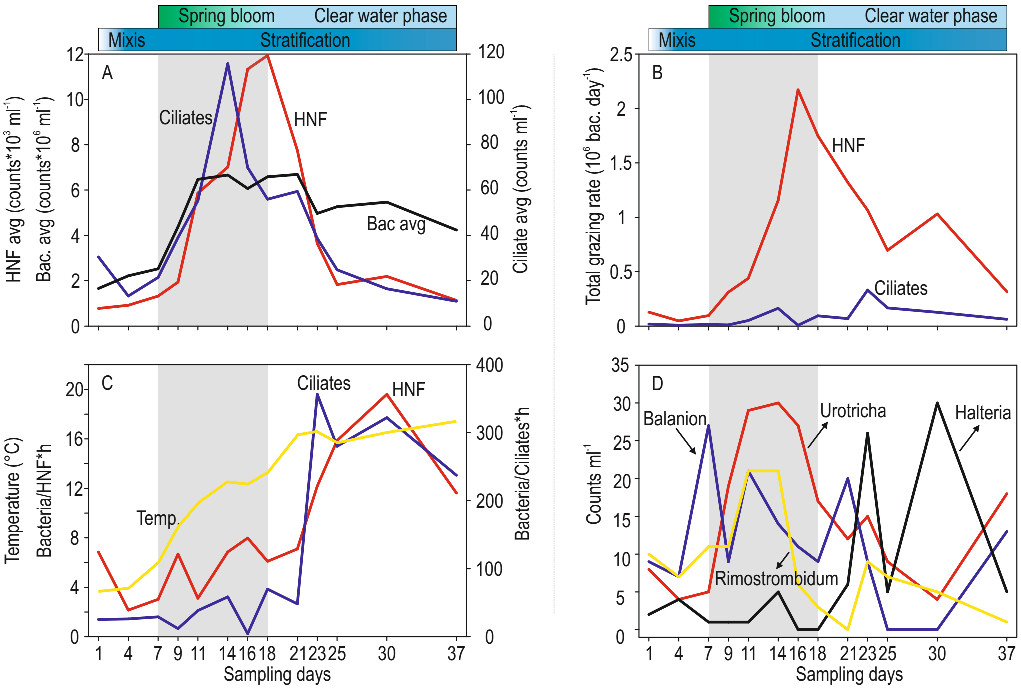

Figure S2. Heterotrophic nanoflagellates and ciliate counts, bacterial ingestion, grazing rate estimations and turnover of observed ciliates in epilimnion. (A) HNF (10^3 ml^{-1}), Bacteria(10^6 mlFigure S3.), and ciliate (per ml) abundances, (B) Total grazing rates of heterotrophic nanoflagellates (HNF) and ciliates, expressed as number of ingested bacteria/day, (C) Bacteria ingested by HNF (per hour) and ciliates(per hour). A temperature scale is also shown (D) Counts (per ml) of four ciliates identifiable by morphology. The grey region indicates the spring bloom as identified by chlorophyll content.

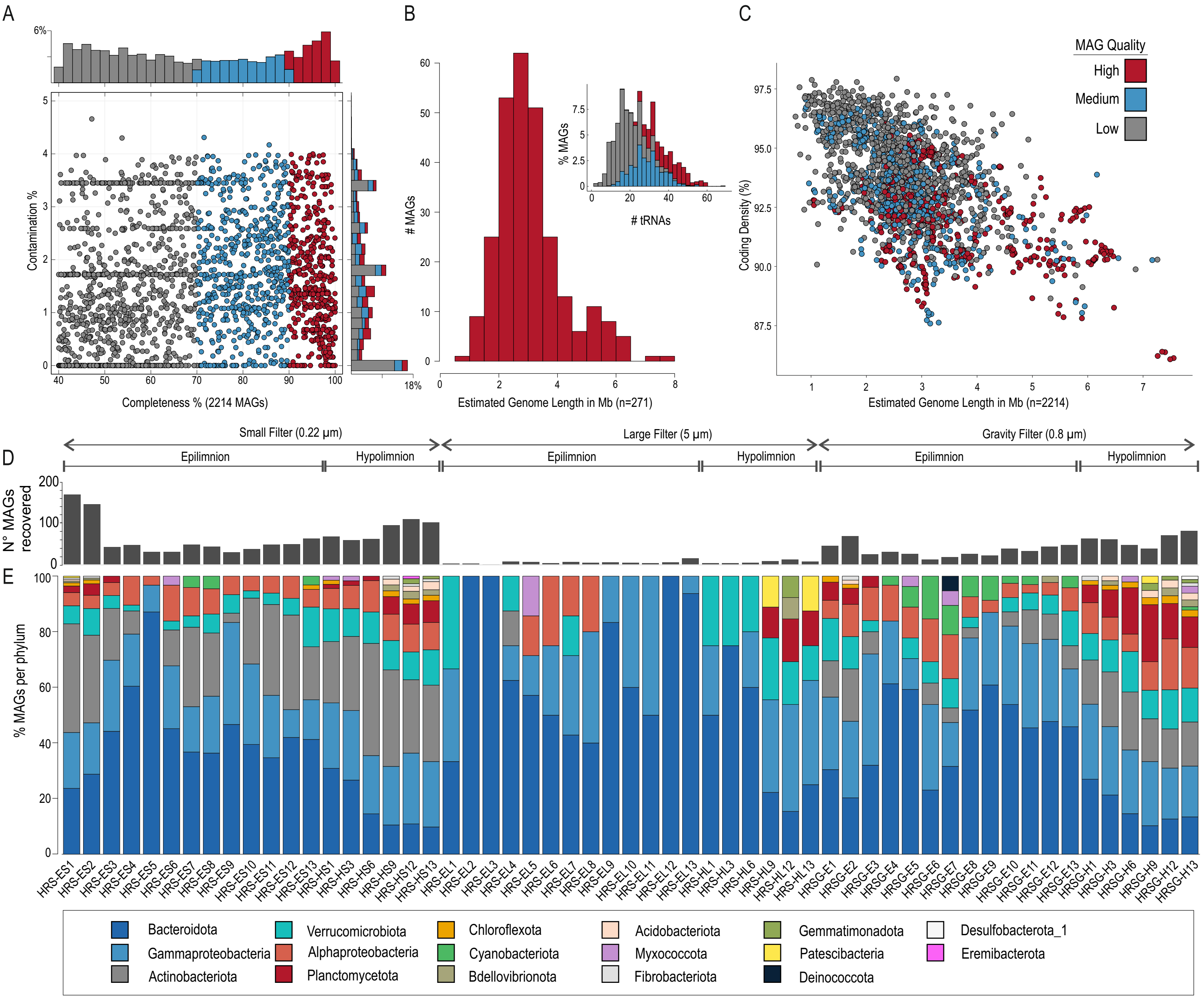

Figure S3. (A) Completeness and contamination estimates for 2214 genomic bins recovered in this study. Three categories of genomic bins were defined based on completeness estimated by CheckM: high quality genomic bins (completeness ≥ 90 %) representing 22.6 % (n = 502) of total, medium quality genomic bins (completeness ≥ 70-89 %) amounting to 26.8 % (n = 594), and partial genomes (completeness 40-70 %) the remaining 50 % (1118). Bins with contamination > 5 % and completeness < 40 % were not analyzed. The histogram parallel to the x axis shows the percentage distribution of bins according to level of completeness while the one parallel to the y axis indicates the percentage distribution of genomes by contamination. (B) Percent distribution of bins according to tRNA gene copy content. (C) Relationship between gene coding density and estimated genome length. (D) Number of MAGs recovered from each dataset (n=57). (E) MAG percentage distribution across phyla.

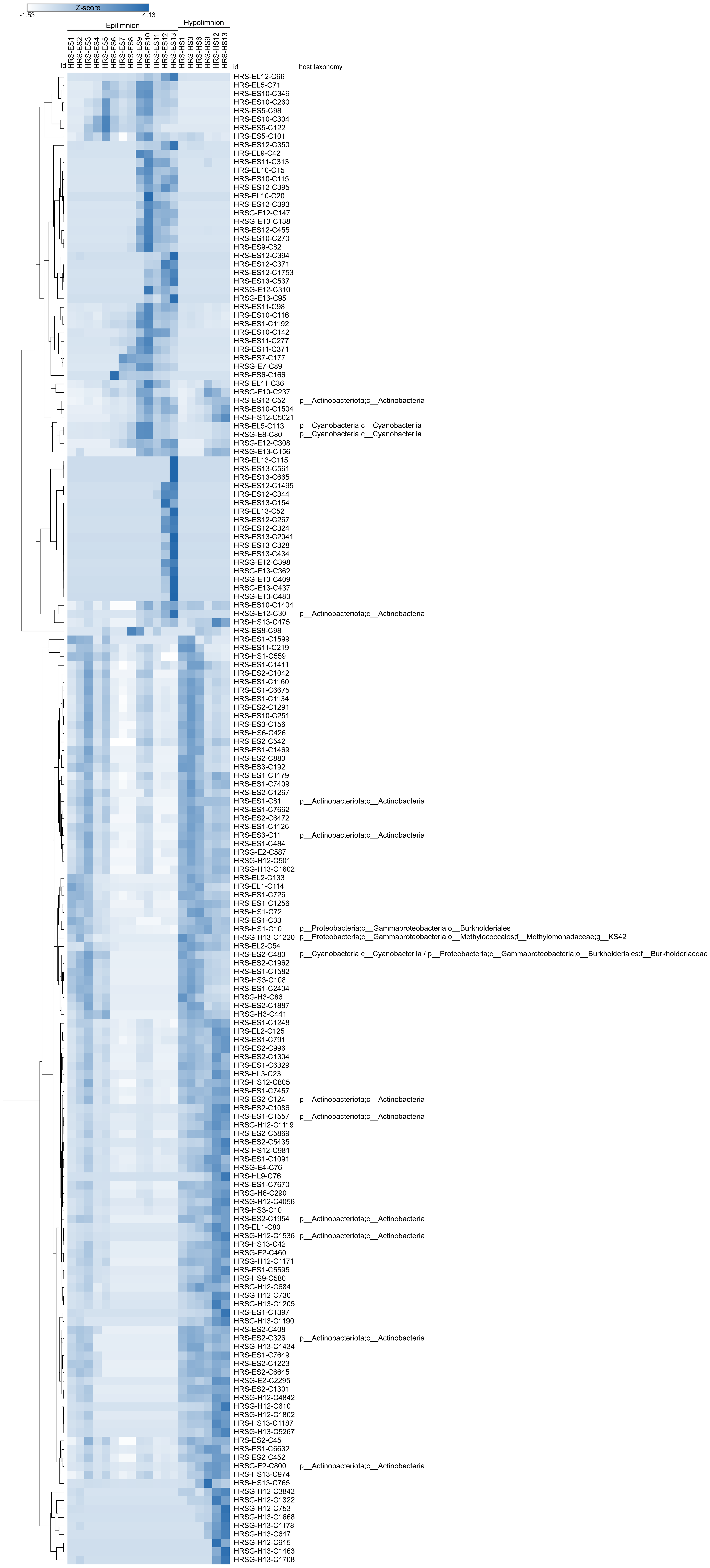

Figure S4. Dereplicated bacteriophages genome abundances of 0.22µm filter (Cov/Gb) normalized by Z-score in epi and hypoliminon (n=175). The rows are clustered by relative abundance by average linkage (with Spearman Rank correlation method). Inferred host taxonomy is shown at the right wherever applicable

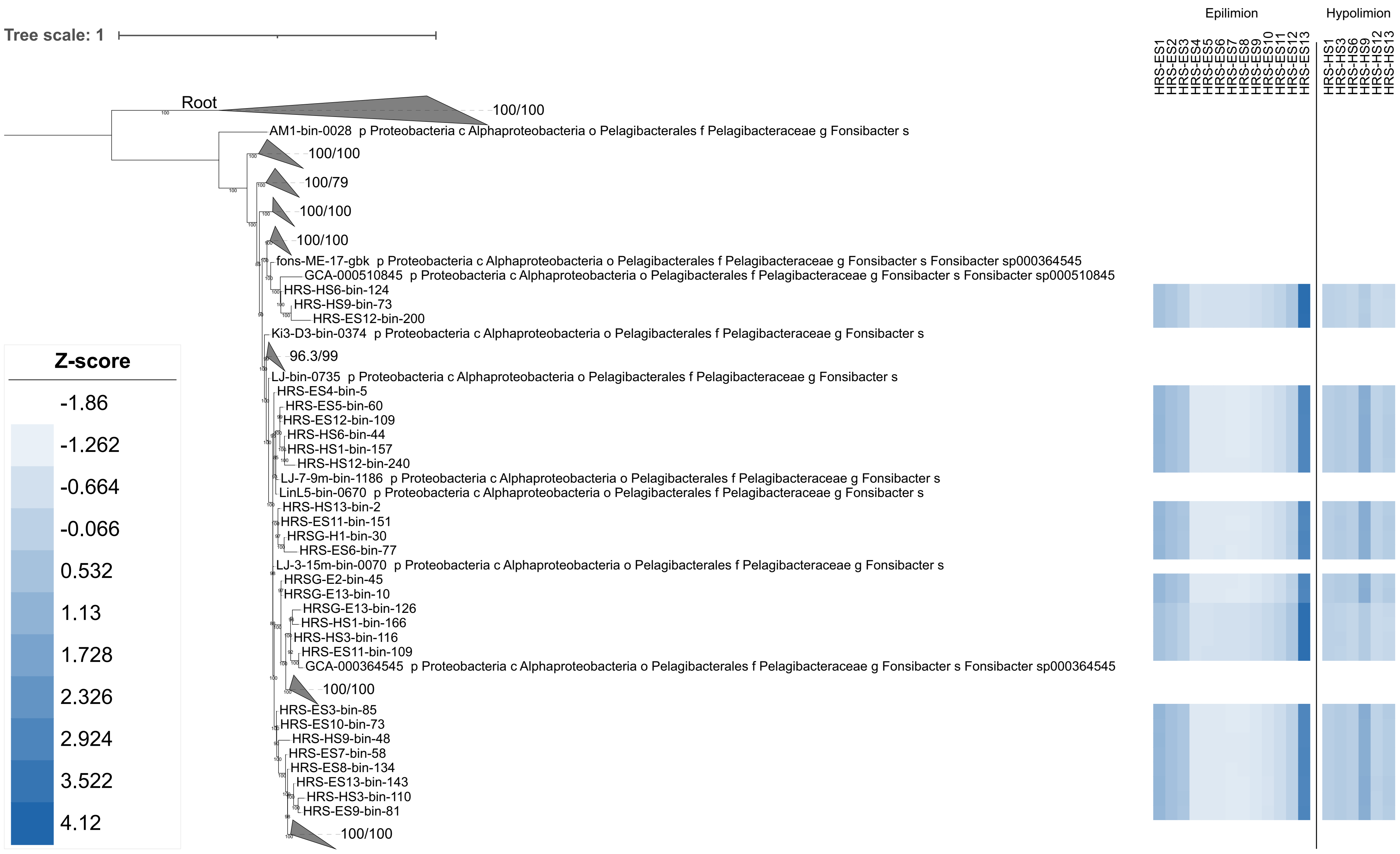

Figure S5. Pruned phylogenomic tree of Ca. Fonsibacter (Pelagibacteriaceae) metagenome assembled genomes. Relative abundance of selected MAGs during the spring bloom in the small filter (0.22µm) are shown on the right side. Each row was normalized by Z-score and clustered by average linkage (with Spearman Rank correlation method). Ultrafast bootstrap values are shown at each node

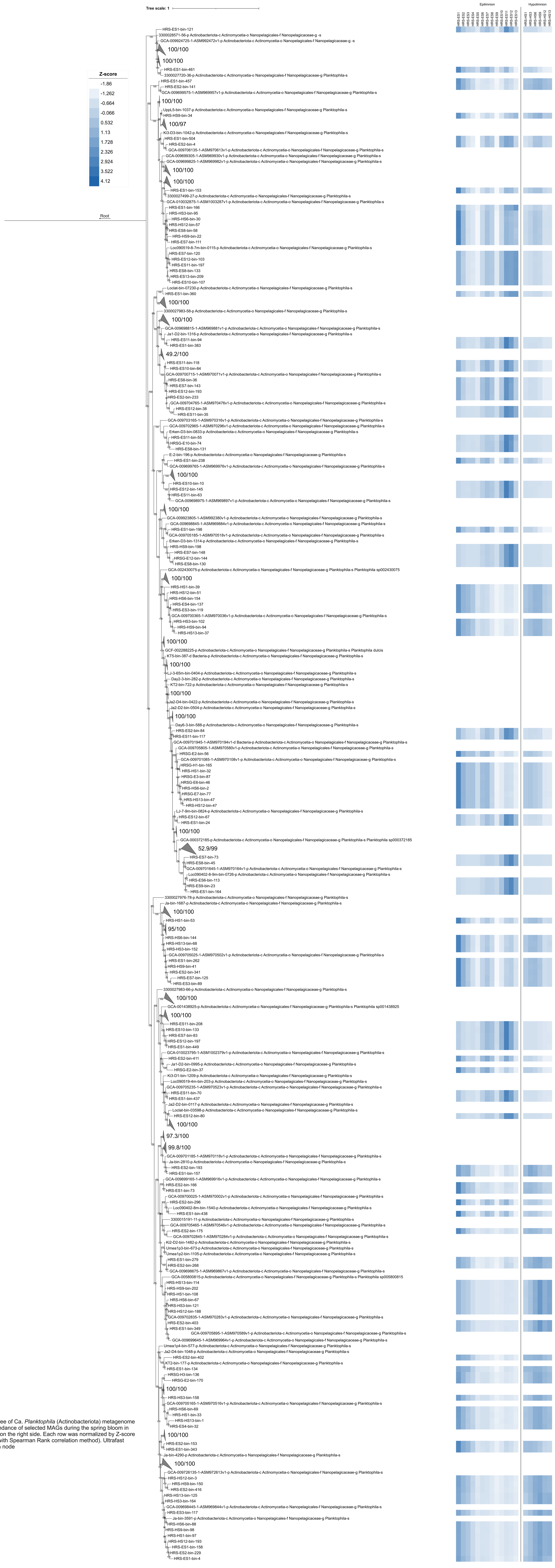

Figure S6. Pruned phylogenomic tree of Ca. *Planktophila* (Actinobacteriota) metagenome assembled genomes. Relative abundance of selected MAGs during the spring bloom in the small filter (0.22µm) are shown on the right side. Each row was normalized by Z-score and clustered by average linkage (with Spearman Rank correlation method). Ultrafast bootstrap values are shown at each node

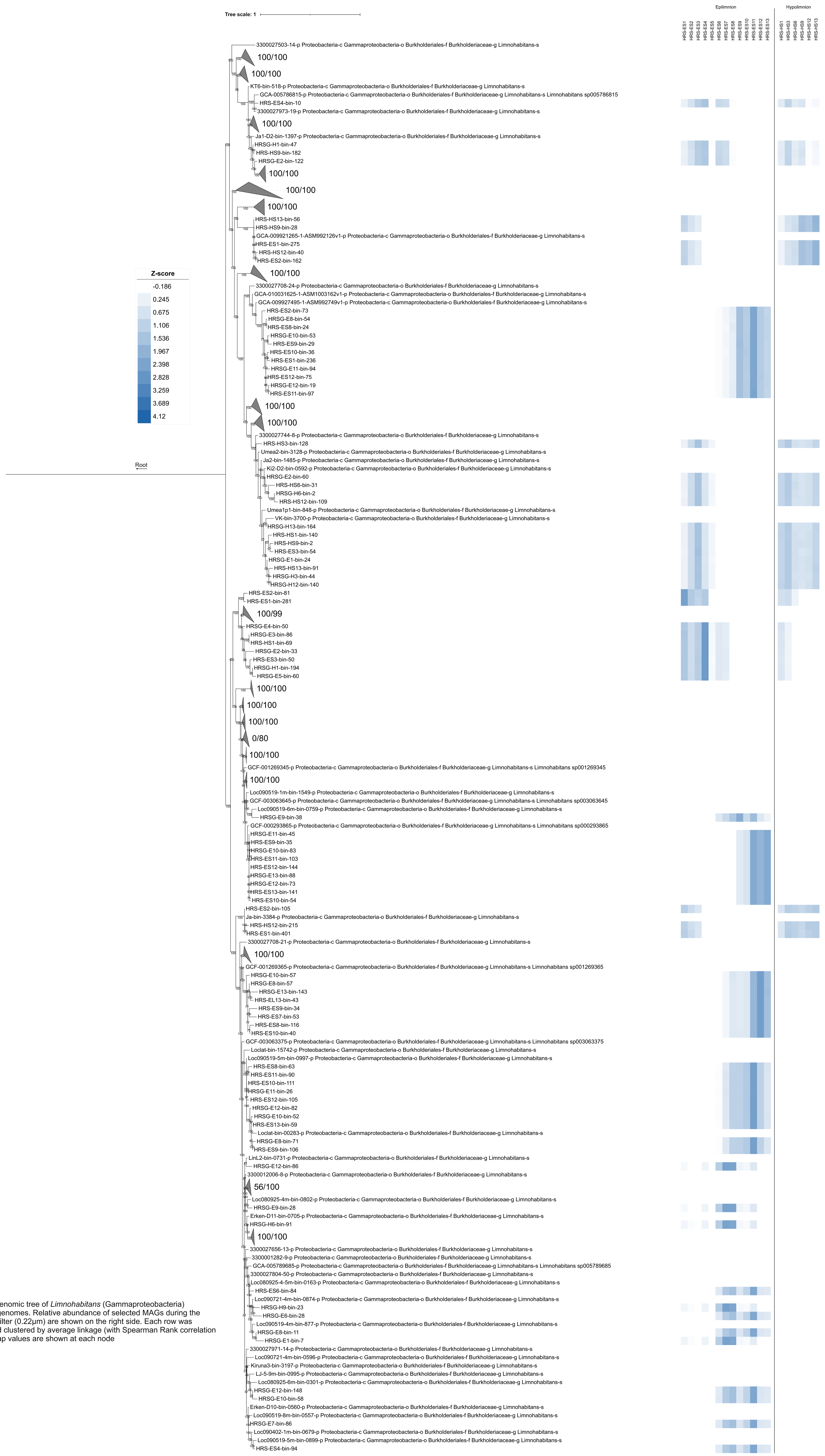

Figure S7. Pruned phylogenomic tree of *Limnohabitans* (Gammaproteobacteria) metagenome assembled genomes. Relative abundance of selected MAGs during the spring bloom in the small filter (0.22µm) are shown on the right side. Each row was normalized by Z-score and clustered by average linkage (with Spearman Rank correlation method). Ultrafast bootstrap values are shown at each node

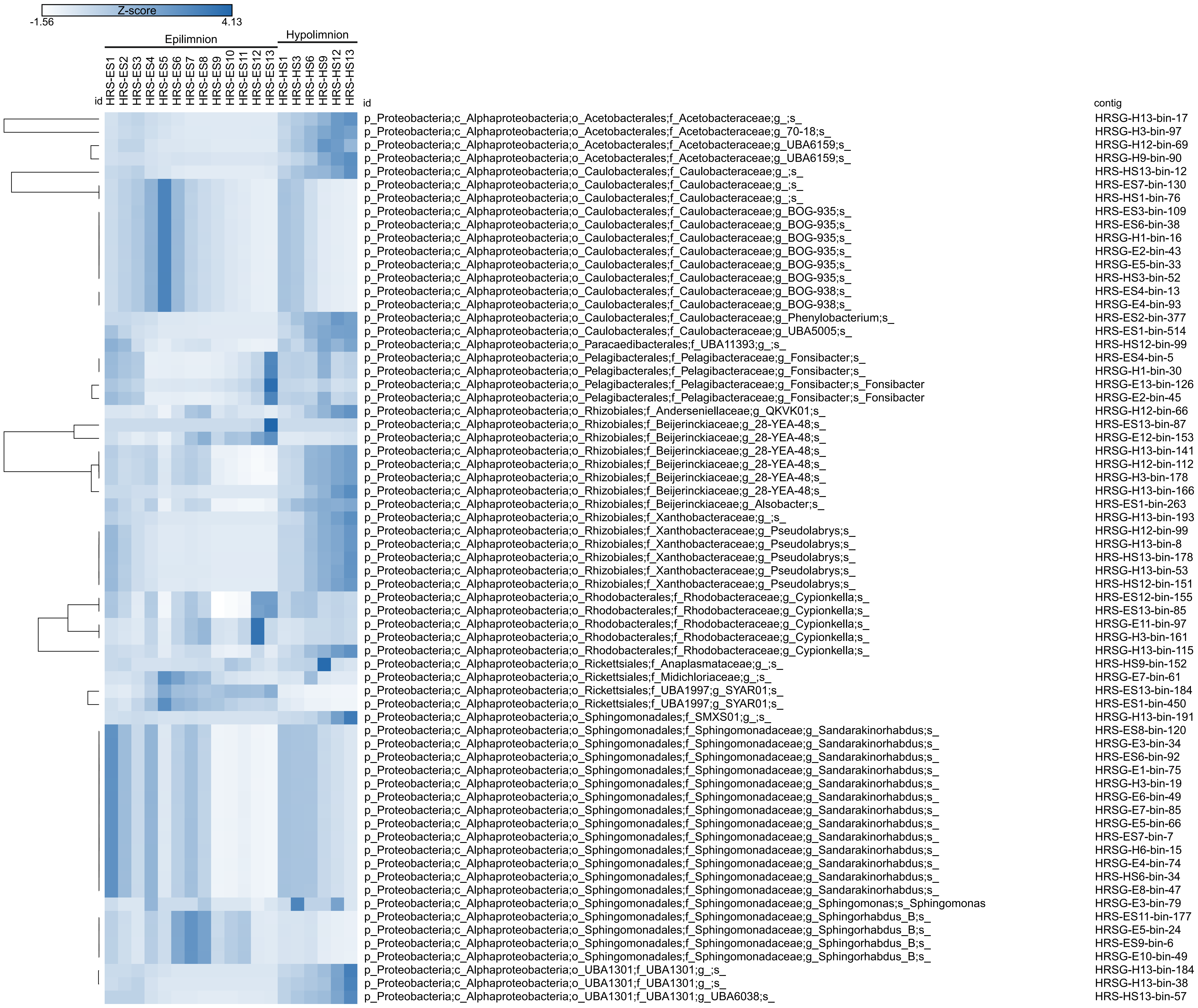

Figure S8. *Dereplicated* Alphaproteobacteria (Proteobacteria) MAG abundances of 0.22μm filter (Cov/Gb) normalized by Z-score in epi and hypoliminon (n=67). The rows are clustered by taxonomy and relative abundance by average linkage (with Spearman Rank correlation method).

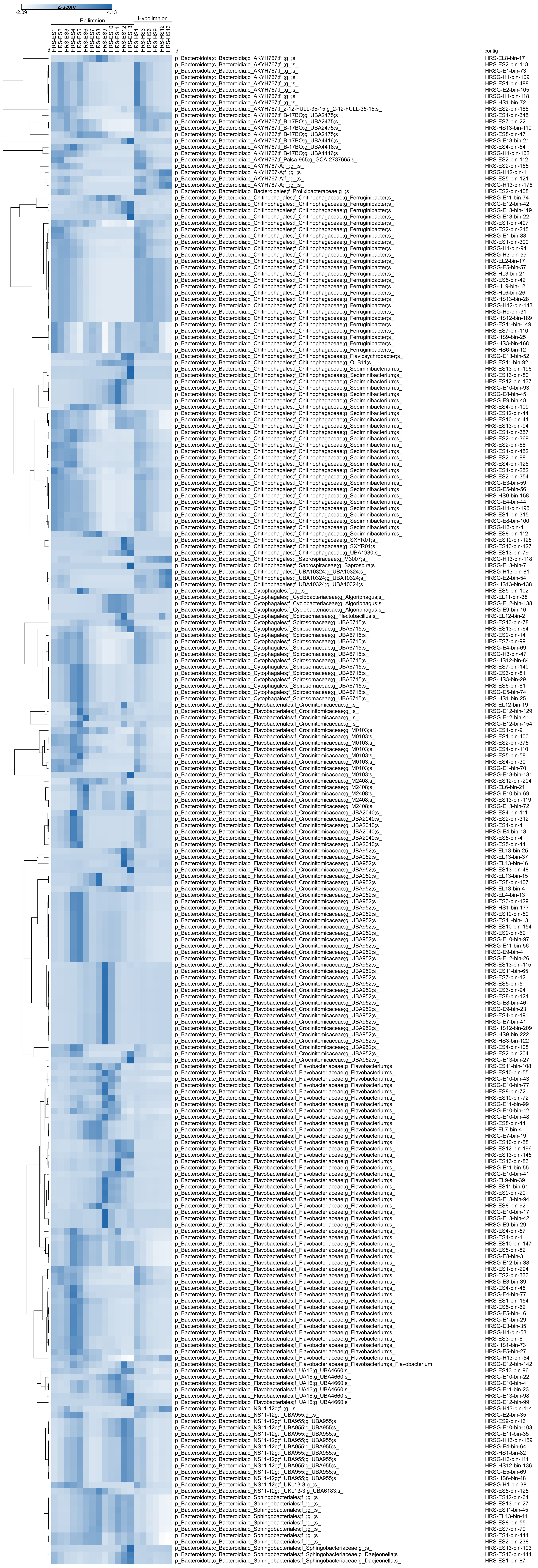

Figure S9. Dereplicated Bacteroidota MAG abundances of 0.22µm filter (Cov/Gb) normalized by Z-score in epi and hypoliminon (n=238). The rows are clustered by taxonomy and relative abundance by average linkage (with Spearman Rank correlation method).

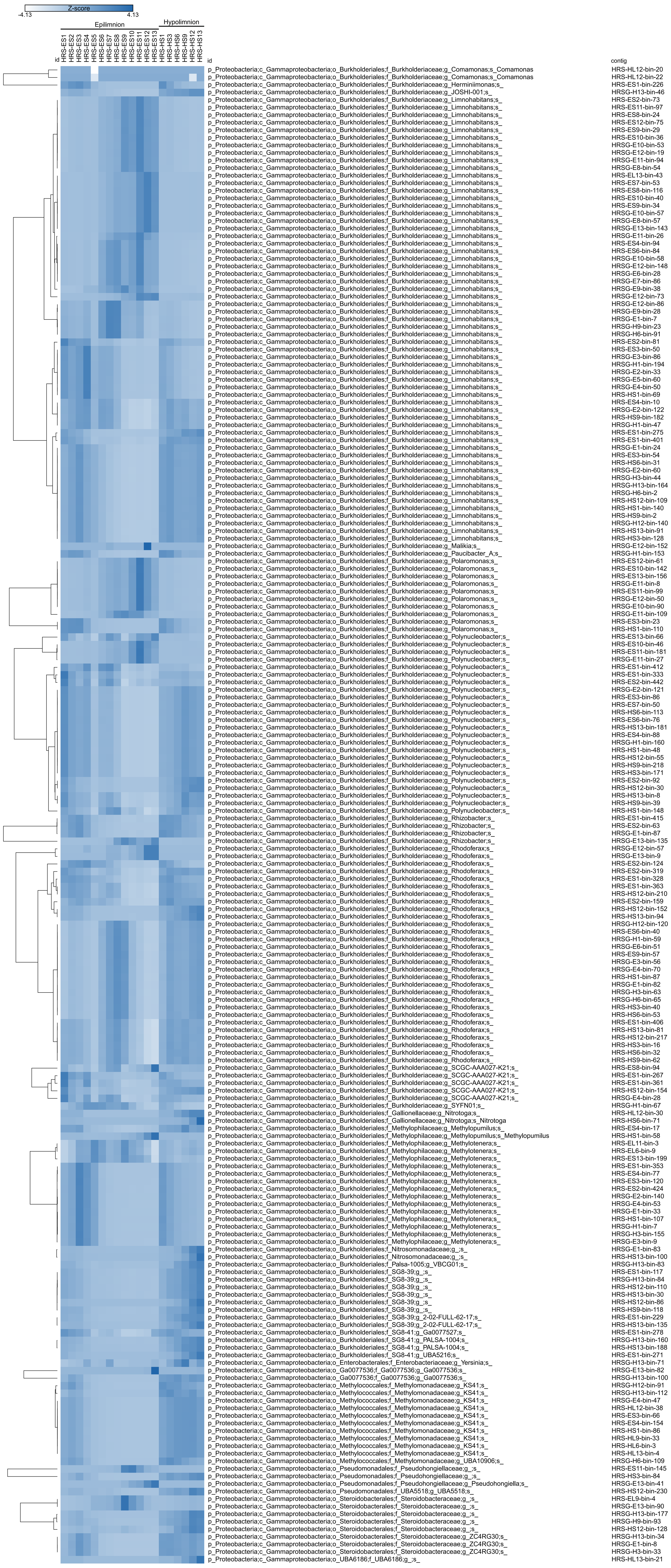

Figure S10. Dereplicated Gammaproteobacteria (Proteobacteria) MAG abundances of 0.22μm filter (Cov/Gb) normalized by Z-score in epi and hypoliminon (n=198). The rows are clustered by taxonomy and relative abundance by average linkage (with Spearman Rank correlation method).

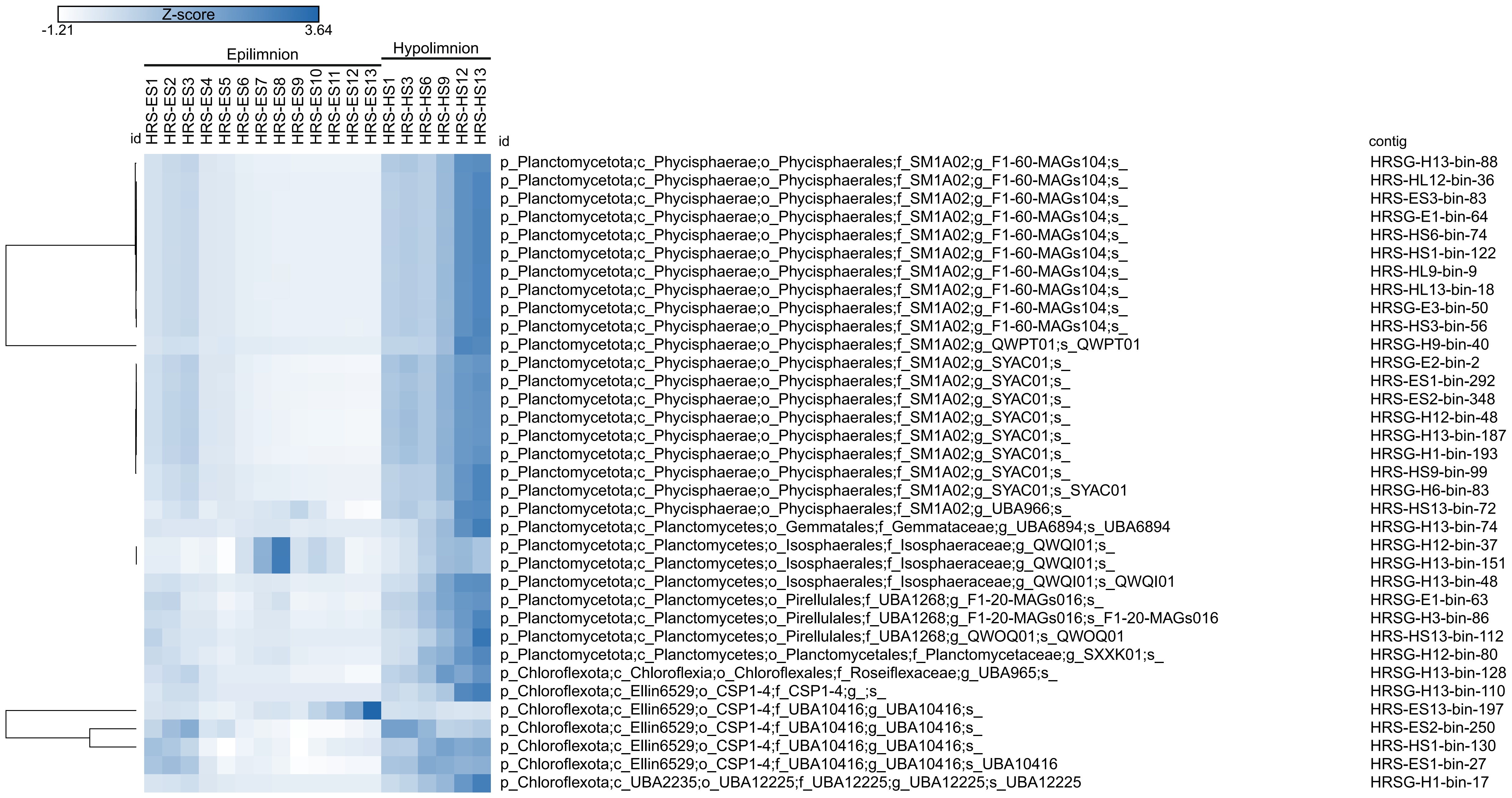

Figure S11. Dereplicated Planctomycetota and Chloroflexota MAG abundances of 0.22µm filter (Cov/Gb) normalized by Z-score in epi and hypoliminon (n=28 and 7,respectively). The rows are clustered by taxonomy and relative abundance by average linkage (with Spearman Rank correlation method).

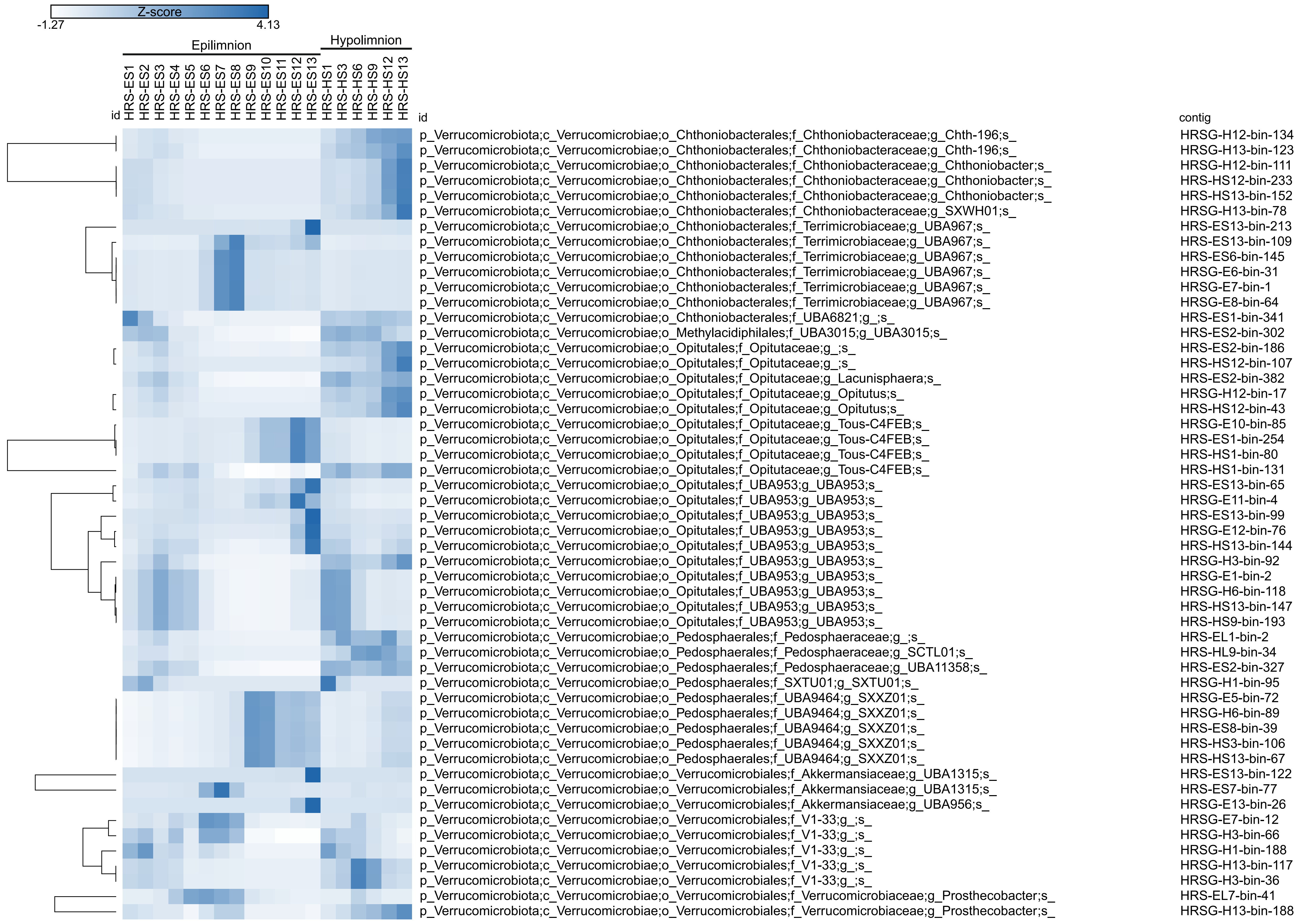

Figure S12. Dereplicated Verrucomicrobiota MAG abundances of 0.22µm filter (Cov/Gb) normalized by Z-score in epi and hypoliminon (n=52). The rows are clustered by taxonomy and relative abundance by average linkage (with Spearman Rank correlation method).

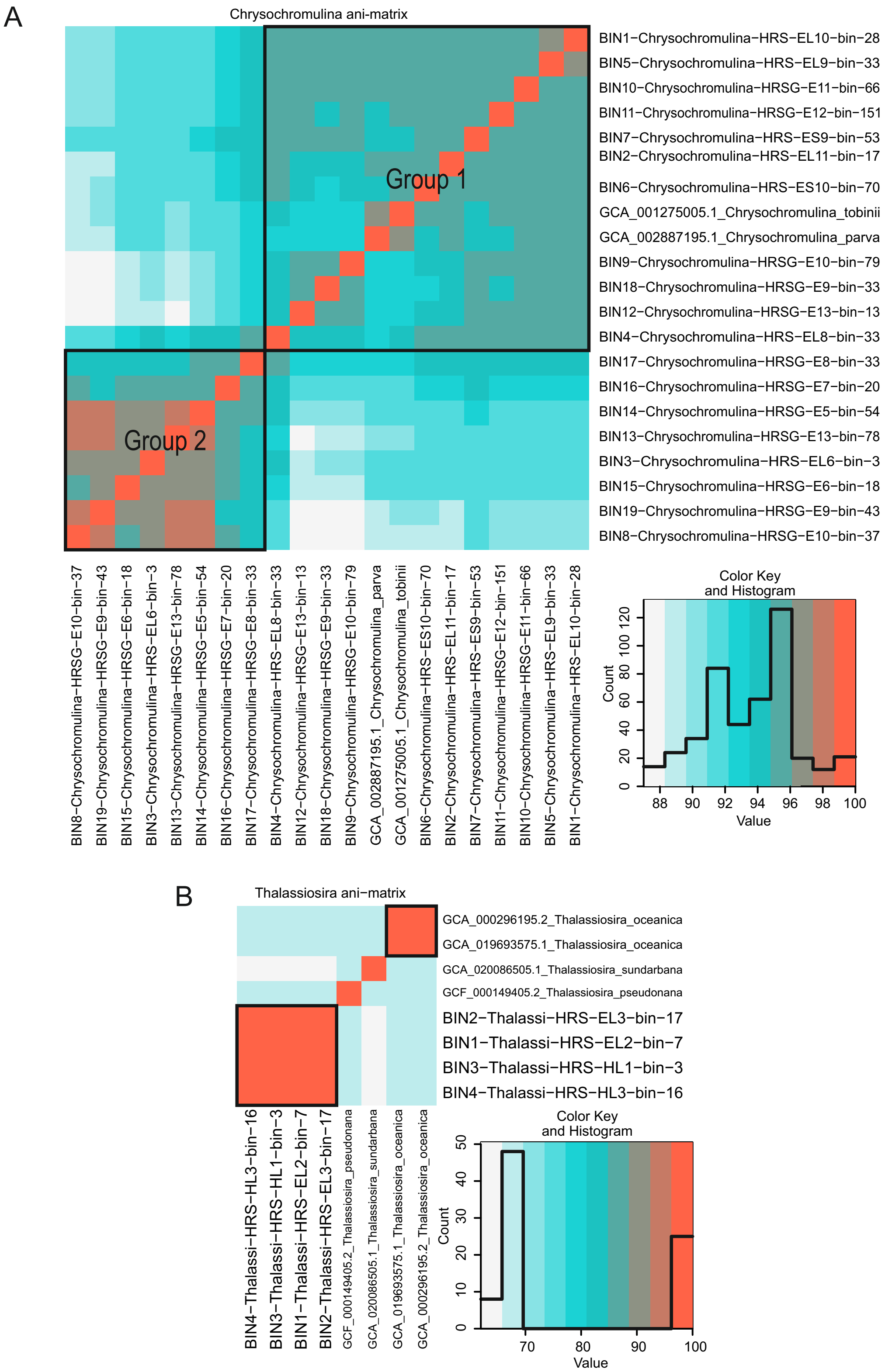

Figure S13. Average Nucleotide Identity (ANI) matrix of (A) *Chrysochromulina* bins, (B) *Thalassiosira* bins. The color key and a histogram of frequency of identity is shown on the left side. Black squares inside the matrices highlight the groups formed on each matrix.

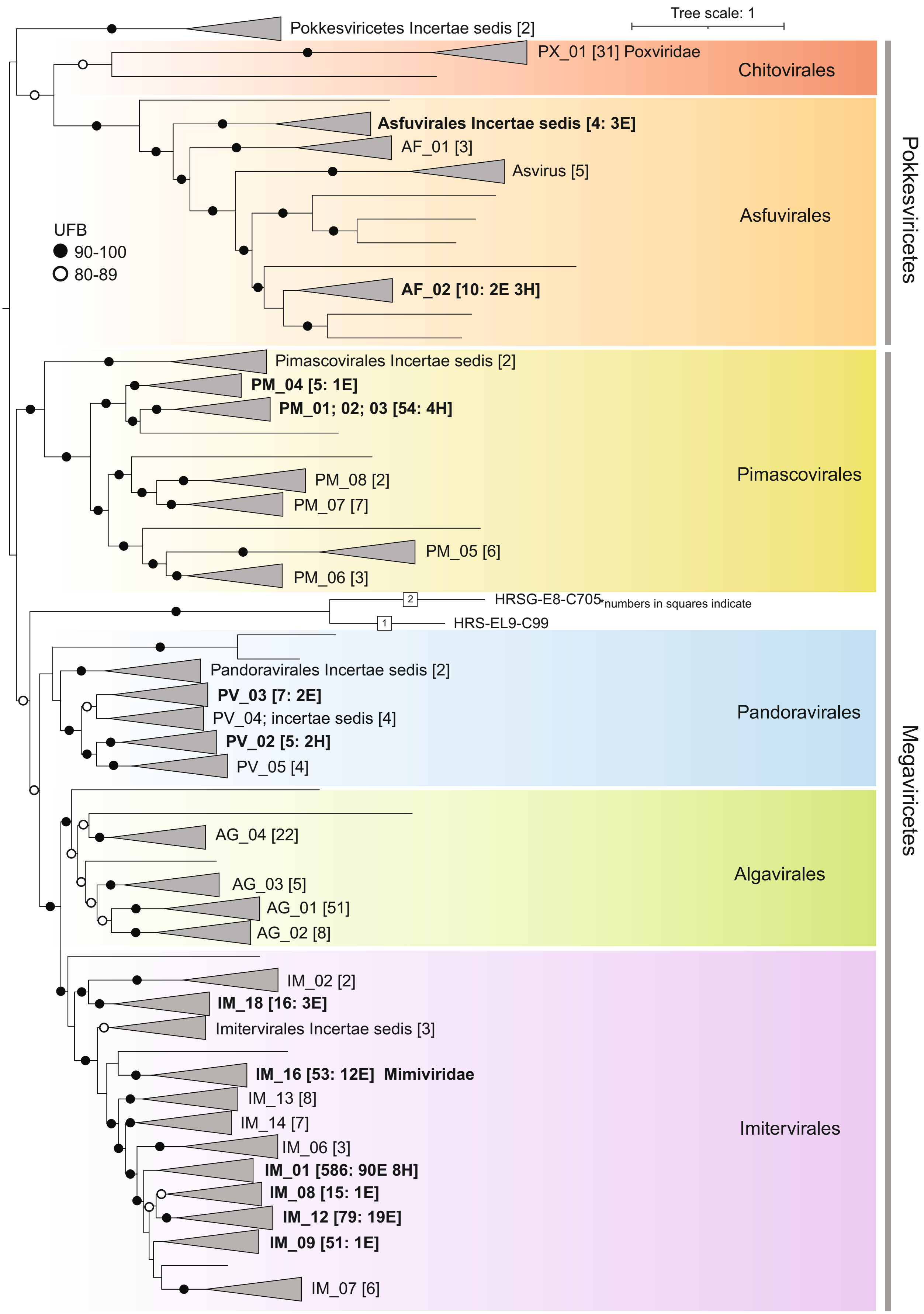

Figure S14. Phylogenomic tree of Nucleocytoviricota based on 7 conserved protein markers. Clades were collapsed at Family level while colored rectangles highlight affiliations to different Orders. Family identifiers are provided on the right of each collapsed clade (triangles) followed by square brackets containing the total number of members within the clade (references + genomes from this study) and by the number of dereplicated genomes recovered here from Epilimnion (E) or Hypolimnion (H) if they are present at all. Ultrafast bootstrap values (UFB) are defined by full (90-100) and empty (80-89) circles. UFB values below 80 are not represented. The root was defined between classes Pokkesviricetes and Megaviricetes as in a previous study (Aylward et. al, 2021).

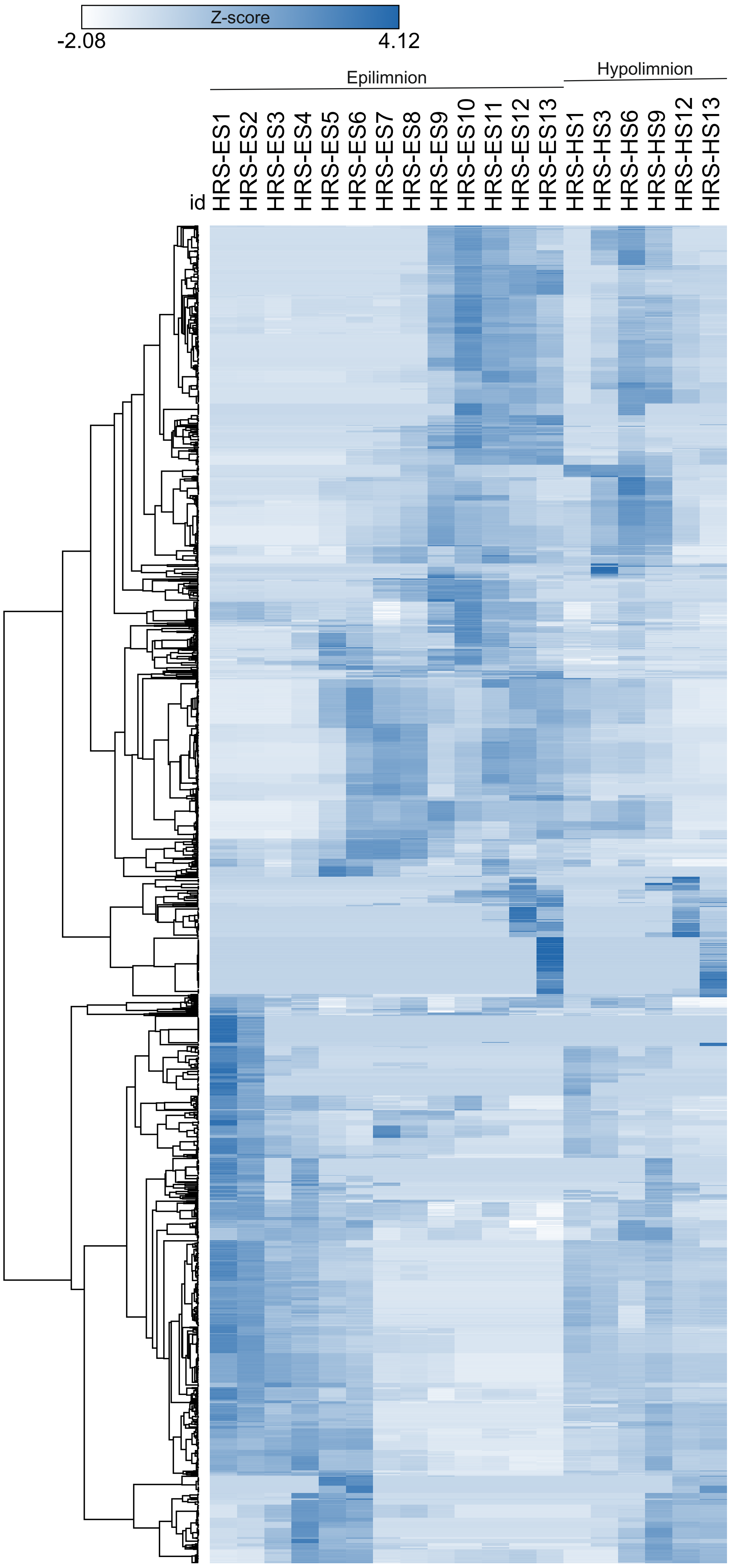

Figure S15. Dereplicated NCLDV contigs cumulative abundances of 0.22, 0.8 and 5 µm filters (Cov/Gb) normalized by Z-score in epi and hypoliminon (n=1721). The rows are clustered relative abundance by average linkage (with Spearman Rank correlation method).

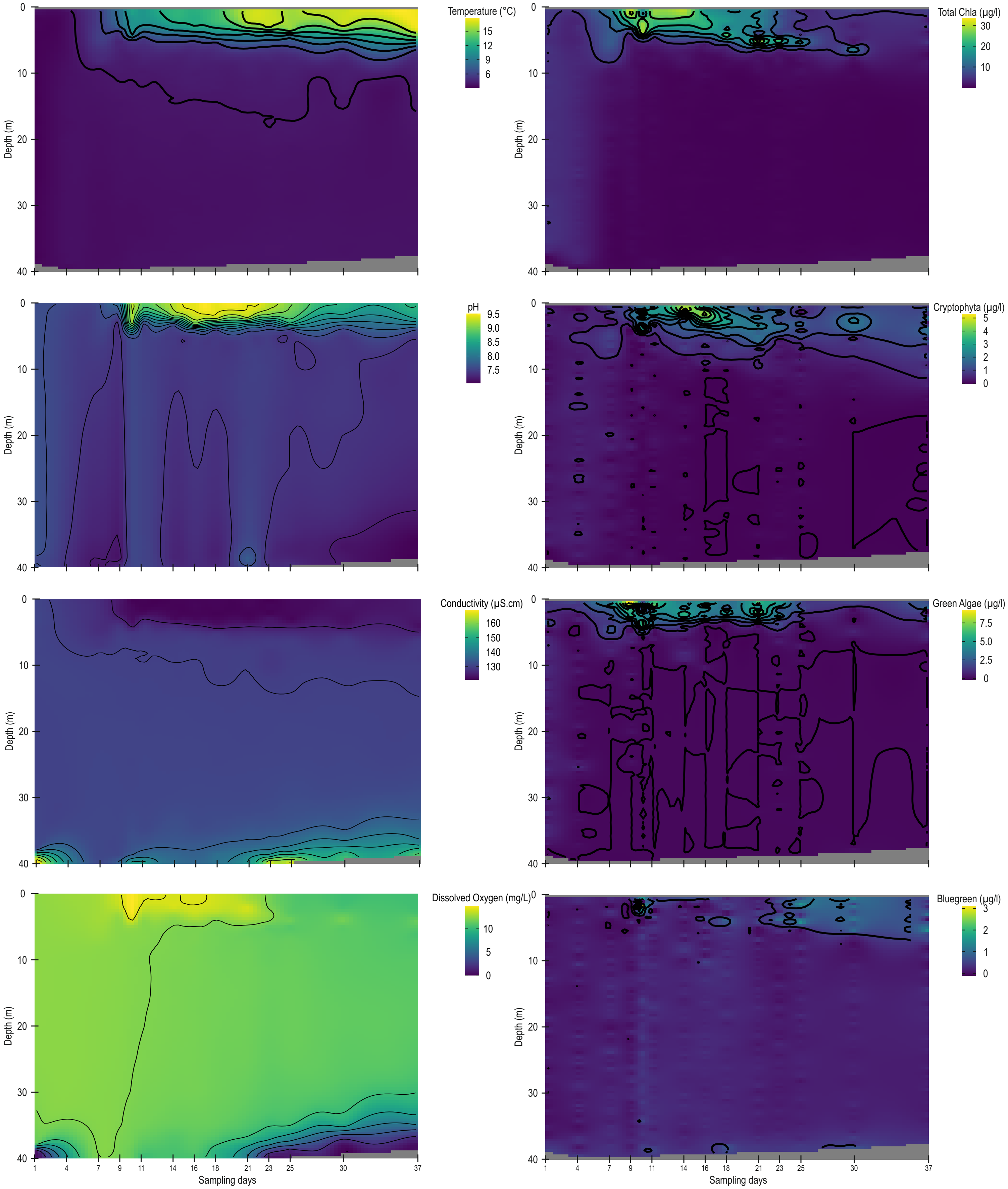

Figure S16. Limnological parameters in Římov reservoir along the spring bloom (03 April ~ 09 May 2018)

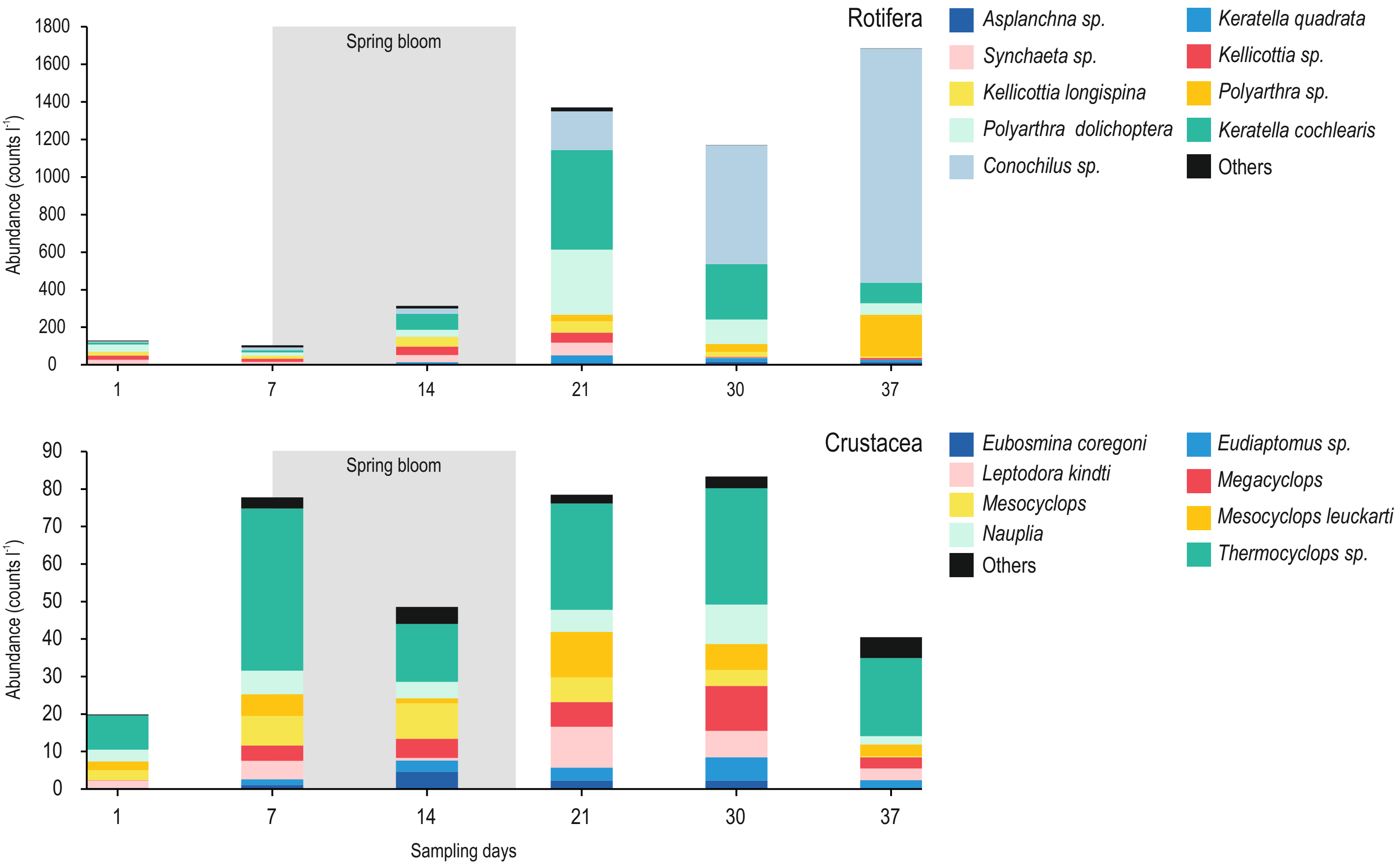

Figure S17. Total counts of zooplankton during the spring bloom. Rotifera (top) and Crustacea (bottom) abundance. For more details of the sampling scheme please check material and methods. Gray square shown in the background mark the Spring bloom during the time period.

List of additional tables

Additional file 2: Table S1. Microscopy data - Phytoplankton counts
Additional file 3: Table S2. General statistics and information about the bacterial MAGs
Additional file 4: Table S3 - MAGs containing rhodopsins
Additional file 5: Table S4. Bacteriophage general information
Additional file 6: Table S5. FISH counts in epilimnion and hypolimnion
Additional file 7: Table S6. Eukaryotic MAGs information
Additional file 8: Table S7. NCLDV abundances and general information
Additional file 9: Table S8. Details of organellar genomes recovered
Additional file 10: Table S9. Characteristics of phylogenomic trees constructed using IQTREE2
Additional file 11: Table S10 - Public datasets used in Figure 4, pane D
